# Engineering next-generation CAR-NK cells for hematological malignancies: genome editing, synthetic biology, and precision immunotherapy

**DOI:** 10.3389/fgeed.2026.1943068

**Published:** 2026-10-13

**Authors:** Ahmed Reda Bahr, Youssef Basem, Amani Alhejely, Abdel Rahman Alaa, Saifallah Mabrouk Abozeid, Amira Basem, Mohsen A. Khormi, Mabrouk A. Abo-Zaid

**Affiliations:** 1 Faculty of Medicine, Alexandria University, Alexandria, Egypt; 2 Medical and Pharmaceutical Industrial Biotechnology Department, College of Biotechnology, Misr University for Science and Technology (MUST), Giza, Egypt; 3 Biology Department, Darb University College, Jazan University, Jazan, Saudi Arabia; 4 Faculty of Medicine, Cairo University, Kasr AlAiny, Egypt; 5 Department of Biotechnology and Genetic Engineering, College of Science, Helwan National University, Cairo, Egypt; 6 Department of Biology, College of Science, Jazan University, Jazan, Saudi Arabia

**Keywords:** cancer immunotherapy, CAR-based immunotherapy, CAR-NK cells, CRISPR genome editing, synthetic biology

## Abstract

Chimeric antigen receptor (CAR)-based immunotherapy has transformed cancer treatment by enabling engineered immune cells to selectively recognize and eliminate malignant targets, with CAR-T cells establishing the first major clinical success in this field. However, severe associated toxicities, logistical complexities of autologous manufacturing, and high rates of antigen-loss relapse have accelerated the development of alternative effector platforms such as CAR-NK cells. CAR-NK cells combine antigen-directed cytotoxicity with the innate antitumor functions of natural killer cells, offering a potentially safer and more readily available off-the-shelf therapeutic strategy. Nevertheless, the clinical efficacy of CAR-NK therapy is constrained by key biological and translational hurdles: brief *in vivo* persistence, inadequate trafficking and solid tumor infiltration, severe susceptibility to tumor microenvironment-mediated immunosuppression, and antigen escape driven by tumor heterogeneity. These biological constraints are compounded by technical bottlenecks, including cell source variability, limited large-scale expansion capacity, *ex vivo* functional exhaustion, and an absence of standardized manufacturing protocols. To address these multi-faceted barriers, recent advances in genome engineering, systems biology, and computational modeling provide promising routes forward. Given that prolonged exposure to immunosuppressive niches induces functional impairment, genome-editing strategies targeting inhibitory checkpoints and persistence programs are crucial; however, enhancing persistence requires stringent safeguards to mitigate toxicity risks from constitutive signaling or genomic instability. Thus, engineering next-generation CAR-NK cells necessitates balancing extended longevity, potent cytotoxicity, and safety. Multiomics approaches facilitate deeper characterization of CAR-NK functional states and tumor–immune interactions, whereas artificial intelligence–guided engineering integrates high-dimensional biological and manufacturing data to optimize receptor design, predict therapeutic performance, and accelerate translational workflows. Collectively, these innovations support a paradigm shift toward programmable CAR-NK cells that can dynamically sense, adapt, and respond to tumor evolution, driving the transition toward autonomous, highly scalable, and precisely controllable cellular immunotherapies in precision oncology.

## Graphical Abstract

**Figure:**
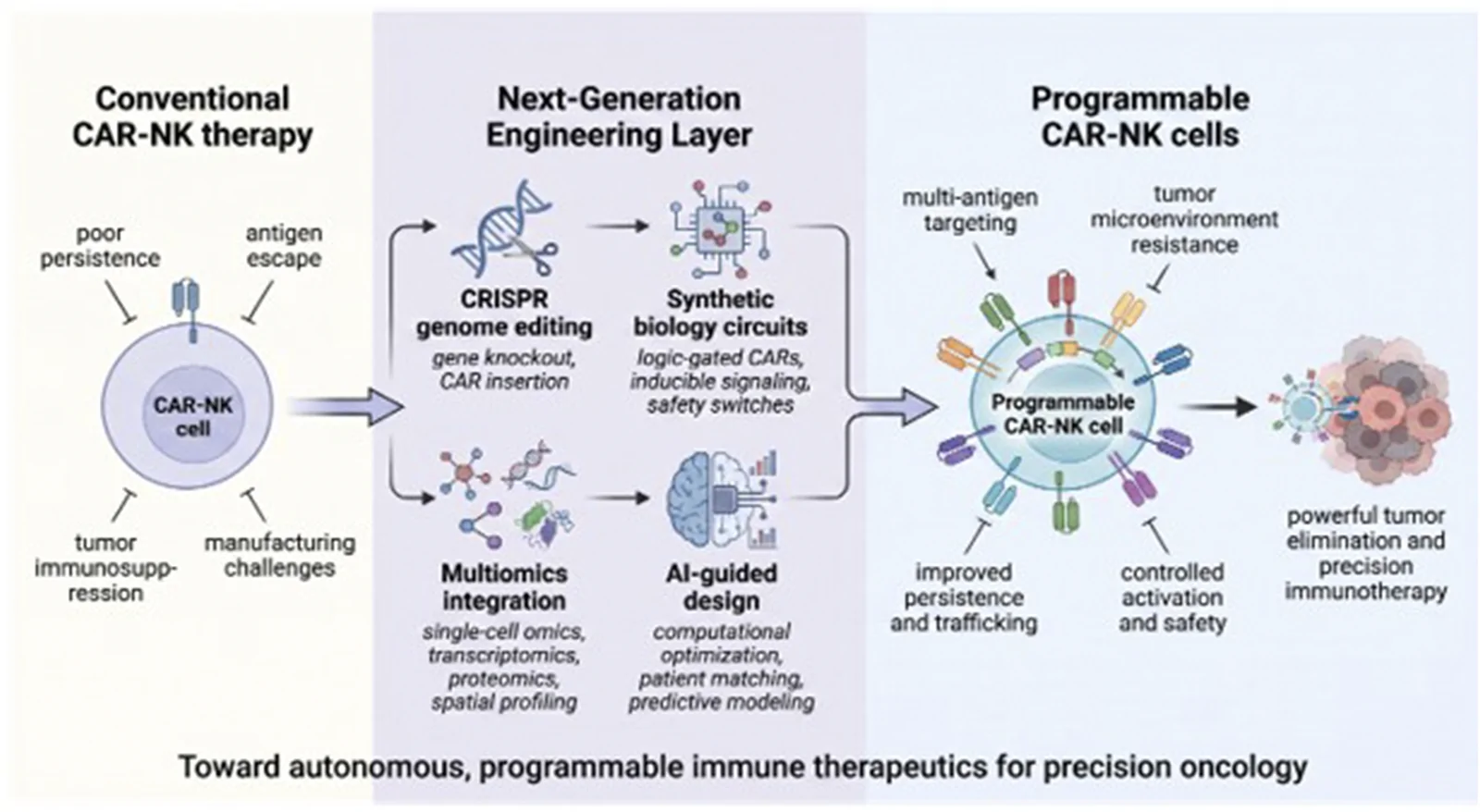

## 1 Introduction

The shift from empirically transferring immune cells towards a more logical approach in programming cells for immunotherapy will take place in hematologic malignancies. This program includes encoding effector identity, receptor specificity, persistence, trafficking, and safety into the design of the immune-cell product before the patient receives it. CAR-NK cells are seen as the next-generation of product platforms because they offer innate cytotoxicity, reversible genetic engineering capabilities, synthetic receptor designs, and scalability for precision immunotherapy (Clubb et al., 2023; Yang et al., 2024a). The conceptual foundation of immune cell therapy stems from allogeneic hematopoietic stem cell transplantation, wherein graft-versus-leukemia activity demonstrated that donor immunity can eradicate malignant hematopoiesis but can also result in graft-versus-host disease and limited control over antigens (Huang et al., 2022). The revolution in CAR T cell technology established that synthetic receptors could be developed to redirect lymphocytes to target lineage-specific antigens such as CD19, BCMA, and others, resulting in deep remissions in patients suffering from all forms of leukemia and lymphoma/myeloma, while exposing relapse mechanisms due to antigen escape, T-cell exhaustion, inflammatory toxicity, and variability in the manufacturing process (Zhang et al., 2022; Huang et al., 2023).

CAR-NK treatment is developing another biological innovation model instead of providing just another, less toxic way in comparison with CAR T to use CAR T as a treatment for cancer. The unique design of the NK cells, as they integrate CAR and endogenous receptors that recognize missing and stressed cells, results in the unique ability of engineered NK cells with CAR constructs to locally destroy or kill malignant cells, regardless of whether they downregulate the expression of primary antigens necessary for the recognition of malignant cells by the T cell immune system or express NKG2D, DNAM-1, or natural killer cell activating receptors for mediated cytotoxicity (Merino et al., 2023). The graft-versus-host reaction from an iPSC-derived CAR-T cell is likely diminished due to the absence of clonotypic T-cell receptors, and both clinical and preclinical evidence indicates that there is a lower incidence of severe cytokine release syndrome and immune effector cell-associated neurotoxicity when using iPSC-derived CAR-T cells compared to conventional CAR-T therapies. However, the durability of the effects of iPSC-derived CAR-T cells has not yet been fully established (Ghobadi et al., 2025). These biological characteristics will also provide a basis for manufacturing models using donor-derived, cord-blood-derived, NK-92, and induced pluripotent stem cell-based products using the same methods to provide off-the-shelf cellular medicines through expansion, standardization, cryopreservation, and distribution of these products in bulk quantities (Goldenson et al., 2022).

The main limitation is no longer redirecting NK cells, but rather whether engineered NK cells will be able to maintain their activity in the hostile environments of hematological systems. The loss of activity *in vivo* is due to cytokine dependence, limited proliferation potential, and tumor-related metabolic failure. One-cell studies have shown that losing the ability to oxidize or convert glucose to energy is a major reason for resistance to CAR-NK therapy and that one part of this issue can be addressed with cytokine engineering (Li L. et al., 2023). Trafficking happens through mechanical means across a variety of cell types, including leukemia, lymphoma, and myeloma, that utilize multiple types of bone marrow and lymphoid niches that are created by CXCL12-rich stroma. CAR-NK cells with no matched chemokine receptors may not be able to traffic effectively to the appropriate sites despite having a high level of cytotoxicity *in vitro*. In order to overcome this spatial barrier *in vivo* between CAR-NK cells and multiple myeloma, BCMA-directed CAR-NK cells were engineered with an increased expression of CXCR4 (Moles et al., 2024). Single-target design limitations, caused by antigen escape, make dual and multiple antigen designs appealing for the treatment of CD19-positive leukemias and lymphomas (Cichocki et al., 2022). The use of shared antigens between normal hematopoietic cells or NK cells increases the potential for fratricide and on-target toxicity. Therefore, the strategy used for selecting and knocking in targets needs to be evaluated and controlled by antigen deletion mechanisms, similarly to what we’ve seen with NK-based therapies targeting CD38 and CD33 (Bexte et al., 2024a; Troy et al., 2024). Cell therapy is transitioning to become engineered cells and now programmable immune systems as a result of these constraints. Through genome editing it is possible to remove inhibitory receptors such as NKG2A and disrupt checkpoint/cytokine-suppressive pathways, as well as insert CARs at defined genomic locations and generate multiplexed edits with minimal dependence upon viral vectors (Bexte et al., 2022; Wang M. et al., 2025).

Instead of using T-cell design, NK-target CAR design is optimizing the signal transduction pathways of NK cells and how they react with other cells that present antigens by using the CD28, CD3ζ, and ZAP70 signaling pathways to fine-tune activation thresholds and cytotoxicity (Acharya et al., 2024; Wang Y. et al., 2024). By using methods such as cytokine armoring, memory-type pre-activation, and integrating cytokine modules at specific sites within the cells, it may be possible to maintain NK cell persistence without generating uncontrolled systemic inflammation (He et al., 2023; Biggi et al., 2024; Zhang Y. et al., 2024). By providing logic-gated receptors, synNotch circuitry, chemokine receptor programming, checkpoint resistance, and molecular profiling-driven patient selection, CAR-NK Therapy is framed as a precise immunotherapy approach whereby the therapeutic product is tailored for the antigen architecture, suppressive ligands, niche chemokines, and the tumour ecology of each patient (Li Y. et al., 2023; Ahmadnia et al., 2024), As shown in Figure 1. CAR-NK therapy has evolved from donor immune-cell transfer and CAR-T therapy into programmable platforms with improved targeting, persistence, safety, and off-the-shelf use.

**FIGURE 1:**
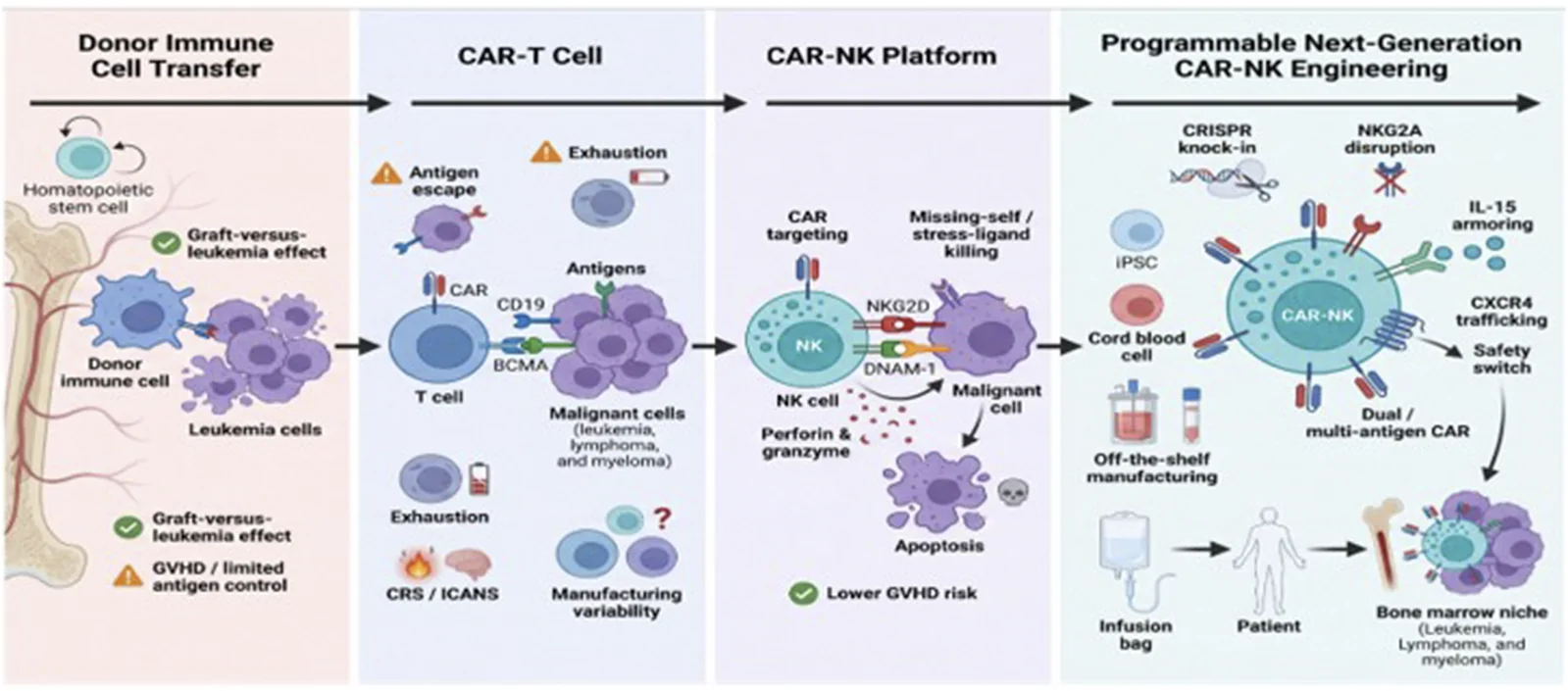
Evolution of CAR-NK engineering platforms, illustrating the shift from donor immune-cell therapy and CAR-T cells to programmable CAR-NK cells for hematologic malignancies.

### 1.1 Literature search strategy

The narrative literature search was conducted using PubMed, Scopus, Web of Science, and Google Scholar for studies published from January 2020 to August 2026. Search terms included combinations of “CAR-NK cells,” “natural killer cells,” “hematological malignancies,” “genome editing,” “CRISPR,” “synthetic biology,” “cytokine engineering,” “iPSC-derived NK cells,” “antigen escape,” “multiomics,” and “artificial intelligence.” Peer-reviewed original studies, clinical studies, and relevant reviews addressing CAR-NK biology, engineering, manufacturing, therapeutic resistance, and translational development were included. Studies unrelated to CAR-NK therapy, duplicate publications, non-peer-reviewed reports, and articles lacking direct relevance to the scope of this review were excluded. CAR-T studies were considered only when they provided directly transferable mechanistic, engineering, manufacturing, or computational insights relevant to CAR-NK development.

## 2 NK cells as an engineering substrate

### 2.1 NK cell biology relevant to engineering

NK (Natural Killer) cells represent a type of innate lymphocyte that functions by killing cancerous or virally infected cells and do not require prior exposure to the antigen to recognize these targets. In contrast to T Cells, NK cells can recognize their target without being restricted by MHC (Major Histocompatibility Complex) through a balance of activating and inhibitory signals received through their receptors. Their natural ability to recognize target cells demonstrates their great potential for genetic engineering since CAR (Chimeric Antigen Receptor) recognition of antigen occurs independently of endogenous receptor signals; thus, NK cells exhibit both CAR and endogenous receptor mechanisms for targeting tumors and minimizing immune escape (Wang W. et al., 2024).

Of all the activating receptors, one of the most studied is NKG2D, as it can recognize stress-induced ligands such as MICA, MICB, and ULBPs that are often overexpressed on tumor cells. Natural cytotoxicity receptors NKp30, NKp44, and NKp46 enhance this recognition of tumors by binding to different ligands that are associated with the presence of cellular stress or malignant transformation. NKp44 is expressed primarily post-cytokine activation; however, NKp30 and NKp46 have a constitutive expression pattern on mature NK cells. Similarly, DNAM-1 (CD226) enhances these pathways through its ability to bind to CD112 and CD155, thereby promoting adhesion between the NK cell and target cells, immune synapse development, cytokine release, and release of cytotoxic granules (Xie et al., 2020).

Inhibitory receptors control NK-cell activation and prevent the autoactivation of these cells. KIRs, or killer-cell immunoglobulin-like receptors, recognize classical HLA class I molecules. Conversely, CD94/NKG2A complex binds to HLA-E to provide a second means of inhibiting NK-cell activation via immunoreceptor tyrosine-based inhibitor motifs (ITIMs). In the tumor microenvironment, immune checkpoint receptors are given increased importance compared to their contribution to NK-cell cytotoxicity, cytokine production, and persistence. For instance, the immune checkpoint receptors of interest in this context are TIGIT and PD-1; TIGIT binds to CD155 and PD-1 binds to PD-L1, thereby inhibiting NK-cell activation through decreased cytotoxicity, cytokine production, and persistence of NK-cells. Therefore, when developing modern CAR-NK cells, the engineering often combines CAR expression with the disruption of checkpoint receptors and/or the rewiring of other receptors to enhance the overall anti-tumor activity of the cells without sacrificing the favorable safety profile that is unique to NK cell-based therapies (Dias et al., 2024).

### 2.2 NK cell functional states

The different functional states of NK cells are the product of their functional plasticity ability to transition between different states based on the presence of cytokines, whether from infection or from their local tumor microenvironment, and multiple types of activation occur. The types of activation that the NK cell can occur by are through activation of NK cells by cytokines. This can occur through IL2, IL12, IL15, IL18 cytokines which activate long-lived NK cells that have undergone multiple rounds of *in vivo* stimulation and thus provide optimal sources for the manufacture of CAR-NK cells. In addition to the long-lived NK cells, the existence of memory-like NK cells created through a brief period of pre-activation by way of cytokines these cells demonstrate extended persistence and thus provide an enhanced anti-tumor effect when subsequently stimulated after being injected back into patients. In this way, there has been a heightened interest in generating NK cells with supportive characteristics that may assist in producing durable and clinically effective CAR-NK cells (Berrien-Elliott et al., 2022).

Cytomegalovirus (CMV) infection often causes the expansion of adaptive NK cells. These cells exhibit unique transcriptional and epigenetic features, allowing for enhanced antibody-dependent cellular cytotoxicity (ADCC), as well as long-term, functional capabilities. On the other hand, exhausted NK cells develop after repeated exposure to the same antigen as well as continuous immunosuppressive signals from the tumor microenvironment, resulting in decreased cytotoxicity, decreased cytokine production and increased expression of inhibitory receptors (i.e., NKG2A, TIGIT, and PD-1). Therefore, limiting NK cell exhaustion has become increasingly focused upon as an engineered approach to preserve activated and memory-like phenotypic traits and thus improve persistence and therapeutic activity (McErlean and McCarthy, 2024).

### 2.3 Sources of CAR-NK cell

Various potential sources of CAR-NK cells have been investigated for CAR-NK cell production, each source ideally expressing different types of biological and clinical benefits. Peripheral blood (PB)-derived NK cells are the most studied source due to their easy availability from healthy individuals and strong cytotoxic ability; however, they possess limited ability to grow in culture (*ex vivo*) and exhibit variability from individual to individual. NK cells derived from umbilical CB are an attractive alternative since they have high proliferation capacity, low risk of infecting patients with latent viruses, and are easily produced in large amounts as allogeneic products produced from multiple donors; however, they typically need cytokine stimulation before they can mature fully functionally (McErlean and McCarthy, 2024).

The advancements made in stem cell technology have allowed for the creation of induced pluripotent stem cell (iPSC)-derived natural killer (NK) cells, allowing for a renewable and highly standardized supply of “off-the-shelf” immunotherapies specializing in NK cell-based therapy. These NK cells allow for precise genome editing prior to differentiation, allowing for uniformity in CAR integration, checkpoint deletion, cytokine engineering, and safety-switch incorporation, all done in a single manufacturing platform. Another commonly utilized platform in NK-92 cell line, which is an immortalized NK cell line that grows robustly, can be easily genetically manipulated, and whose manufacture is reproducible. However, even with these advantages, prior to the clinical use of NK-92 cells, they must undergo irradiation prior to clinical use, as they will engraft, thus limiting the *in vivo* persistence of NK-92 cells. Ultimately, the decision concerning the optimal NK cell source will depend upon the desired balance of scalability, persistence, cytotoxicity potency, complexity of manufacturing, and safety for clinical use (Dehghan et al., 2025).

### 2.4 Engineering opportunities created by NK biology

NK cells have many special biological features that allow them to be an amazing cell type for future engineering efforts. While T cells use antigen loading/Teff receptor (TCR) to recognize target cells, the absence of a TCR on NKs creates a very low probability of developing potential GVHD associated with allogeneic transfer; thus, allowing for universal (off-shelf) CAR-NK product developed manufactured ahead of time, cryopreserved, and used as a common therapeutic for multiple patients without having to collect cells from each person first. This provides greater access to customers while reducing time and cost associated with product development (Wang W. et al., 2024).

In addition, the ability to create multiple gene combinations for those received by NK cells allows for the combination of CAR genes and deletions of inhibitory receptors, the addition of cytokines like IL-15 to support the NK cells, as well as adding “inducible suicide switches” to improve their effectiveness & safety. The acceleration of these engineering methods & the development of standardized gene modifications for NK cells have allowed for better longevity of NK cells, increased antitumor activity, & broader use for treating hematological as well as solid tumors (Schmidt et al., 2022).

### 2.5 Manufacturing standardization and CMC considerations

The development of commercial CAR-NK therapies hinges on the availability of a manufacturing technology that is capable of producing cells in terms of quantity, purity, and biological activity in a regulated and scalable process (Jahan et al., 2024). While K562 cell lines with altered genetics can significantly enhance NK cell production, regulatory authority will require proper assessment of efficiency of K562 cell inactivation process and the absence of any leftover K562 cells in the final product (Jo et al., 2026). Thus, manufacturing of NK cells does require strict regulation of the process, including the regulation of the culturing environment, production parameters, process monitoring, and final specifications of the product (Motallebnejad et al., 2023).

Cryopreservation, therefore, serves as another important component in determining whether CAR-NK products can truly become off-the-shelf therapies: while viability right after thawing does not automatically guarantee post-tissue function (Oyer et al., 2022). Advanced and improved processes for expansion and cryopreservation of the cells result in good cytotoxicity post-thawing, meaning that the freezing conditions and restoring of activity must be taken into account during the process (Lee et al., 2025).

Potency evaluation of cell therapy products may be multi-factorial to include such measurements as live cell counts, therapeutic product expression, genetic modification, and functional bioassays beyond relying on a single analytical output (Simon et al., 2025). In this regard, release testing for NK cell products may account for both identity and purity and viability, sterility, and contaminant control, as well as functional measurements like target cell kill, while product-specific acceptance criteria need to be established in manufacturing development (Von Werz et al., 2025).

The iPSC-derived CAR-NK platforms have paved the way for standardization in manufacturing, as a thoroughly characterized master cell bank serves as a renewable source of genetic material for production purposes (Romanini et al., 2025). Clinical applications of immune cell products from iPSC have proven the feasibility of selecting a specific iPSC clone, developing a master cell bank in accordance with GMP, and qualifying it for its genomic, microbiological, and functional properties (Iinuma et al., 2025).

The production scheme is also affected by the gene-delivery method because the efficiency of lentiviral manipulations will depend on the activities of the vector and the conditions of transduction, which must be kept steady and stable in the course of scaling up (Renner et al., 2024). Non-viral Sleeping Beauty transposition is one more option for getting stable CAR, and, what is important, the production of viral vectors can be minimized, but the processes of transposition should also be kept under control (Bexte et al., 2024b).

### 2.6 Comparison between CAR-T and CAR-NK cells: mechanistic differences, advantages, and limitations of individual platforms

#### 2.6.1 Comparison between CAR-T and CAR-NK cells

CAR-T cell therapy has an extensive clinical track record, with several FDA-approved products and durable, sometimes multi-year, remissions in B-cell malignancies, but it continues to face substantial risks of cytokine release syndrome, immune-effector-cell-associated neurotoxicity syndrome, on-target/off-tumor effects, inconsistent therapeutic responses, and high production costs (Peng et al., 2024; Andrea et al., 2024). By contrast, CAR-NK cell therapy offers several potential advantages, including a markedly lower risk of severe cytokine release syndrome and neurotoxicity, because NK cells predominantly secrete a different cytokine repertoire that is less likely to trigger the systemic inflammatory cascade underlying CRS; NK cells also do not cause graft-versus-host disease, which makes them inherently safer for allogeneic, off-the-shelf use (Balkhi et al., 2025), therefore the comparison between CAR-T and CAR-NK is represented in Table 1.

**TABLE 1:** Comparison between CAR-T and CAR-NK cells.

Feature	CAR-T cells	CAR-NK cells
Clinical track record	Six FDA-approved products; long-term remission data in B-cell malignancies	No approved product yet; multiple early/mid-phase trials showing encouraging responses
Toxicity profile	Substantial risk of cytokine release syndrome (CRS) and ICANS/neurotoxicity, driven by IL-1/IL-6-dominated cytokine release	Low reported CRS/neurotoxicity; NK cells release a different cytokine profile (e.g., TNF-α, GM-CSF) that is less associated with systemic inflammatory toxicity
Graft-versus-host disease (allogeneic use)	Significant risk from endogenous αβ TCR unless disrupted (e.g., TRAC knockout); a major barrier to off-the-shelf products	Minimal risk, since NK cell recognition is not MHC/TCR-restricted, enabling genuinely allogeneic “off-the-shelf” use
Manufacturing model	Predominantly autologous, patient-specific; complex logistics; cost estimated at USD 370,000–530,000 per treatment	Amenable to allogeneic, off-the-shelf manufacturing from PB, UCB, or iPSC sources at potentially lower per-dose cost
Killing mechanism	Strictly CAR/antigen-dependent; antigen-negative escape is a major relapse mechanism	Dual mechanism CAR-directed plus native antigen-independent cytotoxicity (NK receptors, ADCC), reducing antigen-escape relapse
*In vivo* persistence	Generally durable; long-term persistence documented over years in some patients	Major limitation: shorter *in vivo* lifespan and cytokine dependence after infusion
Tumor infiltration (solid tumors)	Variable; established CAR-T infrastructure but still limited in solid tumors	Potentially limited infiltration capacity, compounding persistence challenges in solid tumors
Gene-editing/engineering maturity	Most advanced platform for multiplex editing (Cas9, Cas12a, base/prime editing	Rapidly catching up; multiplex editing and iPSC-derived platforms in active development

CAR-NK cells further combine CAR-directed cytotoxicity with native, antigen-independent killing mechanisms, which can reduce the incidence of antigen-loss relapse that limits CAR-T efficacy, and their off-the-shelf manufacturing feasibility is a distinct practical advantage over the personalized, costly autologous CAR-T workflow (Peng et al., 2024). However, CAR-NK cells present real drawbacks, including a shorter *in vivo* lifespan and potentially more limited tumor infiltration capacity relative to CAR-T cells, both of which currently constrain durable clinical responses, particularly in solid tumors (Andrea et al., 2024).

##### 2.6.1.1 Mechanistic differences, advantages, and limitations of individual platforms

Cas12a is guided to target DNA by a self-processed mature crRNA and recognizes a T-rich PAM motif, forming a more reversible complex with its DNA target than Cas9 (Lei et al., 2024). Cas9 cuts DNA to create blunt ends, while Cas12a makes staggered “sticky” ends that better support precise insertion of the CAR transgene an advantage especially useful for knock-in CAR-NK engineering (Lei et al., 2024). Cas12a shows higher sensitivity to guide RNA mismatches than Cas9, resulting in generally lower off-target recognition and cleavage, although it can still tolerate mismatches distal to the PAM, meaning off-target activity remains a concern as editing efficiency is optimized for therapeutic use (Kim et al., 2020).

Engineered guide chemistries can further reduce this risk: chimeric RNA-DNA (chRDNA) guides for Cas12a support robust editing in primary human T cells with no detectable off-target activity, and an analogous chRDNA approach using Cas9 has been used clinically to engineer the allogeneic anti-CD19 CAR-T product CB-010 (Donohoue et al., 2024). Because both nuclease platforms act via double-strand breaks, they share a genotoxicity risk: Cas9-induced double-strand breaks are associated with large deletions, translocations, chromothripsis, and chromosomal deletions with oncogenic potential, and multiplex editing further increases the likelihood of genotoxicity, translocations, and p53-dependent DNA damage responses (Engel et al., 2025).

This risk is corroborated by direct comparative data: in a head-to-head comparison of CRISPR/Cas9 nuclease editing versus adenine base editing for multiplex allogeneic CAR-T manufacturing, translocations were observed exclusively in Cas9-edited products, whereas base-edited cells achieved higher manufacturing yields and superior *in vitro* effector function under continuous antigen stimulation (Engel et al., 2025). Base editing enables transition mutations (purine-for-purine or pyrimidine-for-pyrimidine substitutions) but cannot perform transversion mutations, and because it avoids double-strand breaks it allows precise single base-pair transitions without inducing genotoxic DSB-associated outcomes (Rossi and Breman, 2024).

Table 2 demonstrates the comparison of genome-editing platforms for engineered cell Therapy: outcomes, advantages, and safety risks. Base editing acts within a narrow specificity window, typically 13–18 base pairs upstream of the PAM; to minimize off-target and bystander effects while preserving efficiency, Cas9 and deaminase variants with altered PAM specificities or editing windows have been developed (Dimitri et al., 2022). Prime editing overcomes the transition-only constraint of base editing by linking a nickase Cas9 to a reverse transcriptase, enabling single base exchanges as well as insertions and deletions of synthetic DNA sequences, but it still suffers from limited editing efficiency and a restricted maximum insert size, with paired-guide prime editing currently allowing insertions of up to approximately 5 kb (Rossi and Breman, 2024).

**TABLE 2:** Comparison of genome-editing platforms for engineered cell therapy: Outcomes, advantages, and safety risks.

Platform	DSB?	Editing outcome	Key advantage	Key limitation/risk
Cas9	Yes	Indels/knockout, HDR knock-in	High efficiency; mature, clinically validated toolset	Translocations, chromothripsis, p53 activation, especially with multiplex editing
Cas12a	Yes	Indels/knockout, sticky-end knock-in	Higher intrinsic mismatch sensitivity; sticky ends favor transgene insertion	Efficiency more target-dependent; residual off-target risk without optimized guides
Base editing	No	Point transitions (C•G→T•A or A•T→G•C)	No DSB; lower genotoxicity; higher manufacturing yield	Transitions only; narrow editing window; possible bystander edits
Prime editing	No (nick only)	Any base substitution; small indels	Greatest versatility without DSBs	Low efficiency; limited cargo size; complex multi-component delivery

As with standard nuclease-based editors, both base and prime editors remain at risk of off-target genomic modification, and the low efficiency of prime editing remains its most significant limitation, requiring further optimization of the nickase Cas9, reverse transcriptase, and pegRNA components (Rossi and Breman, 2024; Moradi et al., 2024). This has motivated delivery innovations such as engineered virus-like particles (eVLPs), which achieve 1.5–2.8-fold improvements in prime editing efficiency across diverse genomic targets and cell types without increasing off-target effects, compromising cell viability, or disrupting gene expression profiles (Langley et al., 2026).

## 3 Genome editing revolution in CAR-NK engineering

Genome editing transforms CAR-NK therapy from the traditional model of transducing a receptor for CAR-NK cells to a more rationally engineered approach to rewriting immune cells. In the treatment of hematologic malignancies, genome editing technologies will be able to improve target specificity and provide persistence, checkpoint resistance, cytokine autonomy, and consistency of manufacture. This is a particularly important transformation because the efficacy of CAR-NK cells is dependent upon the expression of a CAR, but it also requires durable fitness in the marrow and lymphoid niches (Pacesa et al., 2024; Lei et al., 2024).

### 3.1 CRISPR-based engineering platforms

The CRISPR-Cas9 system is the basis for almost all CAR-NK cell genome editing because of its ability to knock-in and/or knock-out nucleotides from the genome as well as provide functional screening. In NK cells, Cas9 nucleases can delete inhibitory receptors, insert synthetic receptors, and disrupt pathways that are responsible for immune suppression in the host. Despite these possibilities, primary NK cell manufacturing has several ongoing challenges, including editing efficiency, cell viability loss, and toxicity due to DNA double-strand breaks (Moradi et al., 2024; Song et al., 2024).

The Cas12a, base editing, and prime editing methods allow for a much larger area of design than only using Cas9’s cleavage ability. In particular, Cas12a can do multiplexed editing through small guide arrays, meaning a collection of different guide RNA sequences. Base editing involves converting nucleotides with precision without making double-strand breaks in DNA. Prime editing can produce an even more extensive range of sequence modifications, but the current ability to deliver and efficiently use prime editing within primary NK cells is not yet as well developed as it is with T cells (Mohr et al., 2023; Petri et al., 2025).

### 3.2 Checkpoint deletion strategies

The goal of checkpoint removal is to ensure that CAR-NK cells do not experience any inhibitory signals from the malignant or stromal compartments. A good example of this is CISH knockout, as CISH acts to inhibit IL-15 signaling and so restrict NK cell proliferation and cytotoxic capacity. The average disruption or functional changes that occur in the NKG2A element can also help to overcome HLA-E-mediated inhibition, which is a key immunosuppressive mechanism used by hematological malignancies (Zhu et al., 2020; Sætersmoen et al., 2025).

TIGIT, PD-1, and LAG-3 are three additional inhibitory axes that can inhibit engineered NK activity following constant contact with the tumor. TIGIT has the ability to decrease the overall strength of an immune synapse, and LAG-3 expression indicates activated but hyporesponsive NK cell states. Editing these pathways may positively affect serial killing; however, indiscriminately removing checkpoints could cause increased exhaustion, tonic activation, or risk for inflammation (Navin et al., 2025; Vasilyeva et al., 2025).

### 3.3 Precision CAR integration

Precision CAR integration addresses the uncertainty surrounding the erratic nature of viral insertions. By utilizing targeted knock-in to specifically defined genomic loci, the amount of variability between CAR expressions within an individual patient can be reduced, as can the risk from insertional mutagenesis and the use of high levels of constitutive signaling due to the uncontrolled copy number of the product. Site-specific integration is especially advantageous with CAR-NK therapy because it allows for a constant, stable potency across multiple large batch manufacturing processes required for off-the-shelf standardized therapeutic products (Dabiri et al., 2023; Kath et al., 2024).

Using a safe-harbor approach or a stem cell-derived approach to create clonal master cell banks with predetermined edits can further help to standardize CAR-NK production. iPSC- or ESC-derived NK cells may allow for the ability to manufacture from the same defined engineered source multiple times while enabling CAR insertion, immune evasion edits, and cytokine modules to be validated prior to final differentiation of NK products (Kwon et al., 2024; Zhang Q. et al., 2024).

### 3.4 Multiplex genome engineering

Multiple genome engineering techniques for creating CAR-NK cells allow for the simultaneous modification of many different aspects of the CAR-NK cells. The CAR-NK cells must have their CARs inserted, their checkpoints deleted, their cytokine support, and their resistance pathways edited in order for the CAR-NK cells to be effective in suppressive hematologic environments. While this is a very powerful mechanism, the addition of each additional edit also increases the risk of viability, genomic instability, and complexity in manufacturing (Bexte and Wagner, 2025; Su et al., 2025).

Multiplex editing has a great deal of power when its edits are coordinated as a function together instead of accumulating separately; e.g., CAR expression may occur concomitantly with checkpoint resistance and cytokine independence, as well as the disruption of apoptotic or metabolic pathways of resistance. High-throughput CRISPR screens and base-edited allogenic will soon help define what edit combinations create improved potency for the ultimate benefit of cellular fitness (Datlinger et al., 2025; Engel et al., 2025).

Importantly, the current evidence supporting these next-generation engineering approaches remains at different translational stages. While several CRISPR-based modifications, synthetic-biology circuits, and AI-guided design strategies have demonstrated promising results in preclinical CAR-NK models, clinical validation remains limited. Most ongoing clinical evidence is currently derived from early-phase CAR-NK trials evaluating engineered constructs, whereas advanced computational optimization and programmable synthetic circuits largely remain investigational and require further experimental and clinical validation (Clubb et al., 2023).

### 3.5 Epigenetic engineering

Nuclease-dead Cas9 (dCas9) can be fused to transcriptional repressors or activators to achieve CRISPR interference (CRISPRi) or CRISPR activation (CRISPRa); CRISPRi typically uses a Küppel-associated box (KRAB) domain to repress transcription at the target start site, while CRISPRa uses activators such as VP64 to promote endogenous gene expression, and dCas9 can also be fused to TET demethylases or DNA methyltransferases to induce locus-specific demethylation or methylation, including at hypermethylated tumor-suppressor promoters (Dimitri et al., 2022). This is particularly relevant to counteracting exhaustion in engineered effector cells: epigenetic editing offers a strategy for precise and durable regulation of CAR-T/NK cell gene expression without altering the underlying DNA sequence, and genome-wide CRISPRa/i screens have been used to map the regulatory circuits governing cytokine expression and cell activation (Horvathova et al., 2026).

A concrete example now cited is the use of transiently delivered “designer epigenome modifiers” simultaneously targeting PDCD1 and LAG-3 via mRNA electroporation a hit-and-run, non-viral approach that avoids the permanent DNA alterations associated with CRISPR-Cas9 knockout while circumventing the need for lentiviral vectors typically required to sustain KRAB-mediated silencing (Horvathova et al., 2025).

Persistent expression of dCas9-based activators or repressors can cause off-target transcriptional perturbations and select for clonal variants with a growth advantage, and the durability of the intended epigenetic change is often unpredictable, being transient in some contexts and stable in others; because NK cells have distinct epigenetic programs and subset-specific homeostasis, these uncertainties require single-cell transcriptomic and ATAC-seq profiling, time-course durability studies, and clonality monitoring during release testing (Dong et al., 2026).

### 3.6 Emerging epigenome editing

The epigenome can be edited in such a way that it provides an additional method of sharing CAR-NK programming in a reversible manner via modified gene expression rather than by modifying the DNA sequence on a permanent basis. CRISPR interference will be able to act as an inhibitory mechanism for inhibiting exhaustion-related genes, while CRISPR activation, on the other hand, will activate and catalyze genes associated with cytotoxicity, trafficking, and persistence. It is possible that these methods could be beneficial in situations where full knockout is not safe or graded control of expression is advantageous *in lieu* of complete epigenome correction (Azeez et al., 2024).

Chromatin modification might prove instrumental for maintaining CAR-NK functional identity post-expansion and after long-term cryopreservation. Transcriptional and epigenetic states are key players influencing NK-cell fatigue, metabolism, and developmental contents during effector potential. Rather than solely targeting receptor deletion events, future modifications may generate stable locations of effector accessibility in conjunction with the suppression of dysfunction or terminally exhausted programs (Chehelgerdi et al., 2024; Khodabandehloo et al., 2025).

### 3.7 Safety challenges

The most important issue regarding the safety of CAR-NK therapy is the possibility of not just off-target mutations but also structural damage to the genome. Double-strand breaks can lead to the generation of deletions, translocations, chromosomal rearrangements, or even complete loss of chromosomes, particularly with multiplex editing. As a result, sensitive assays other than standard off-target predictions are required for assessing potential risk; these include karyotyping, various methods for detecting structural variants across the entire genome, such as linked-read sequencing and optical genome mapping, and long-read sequencing (Lazar et al., 2024; Tsai et al., 2023).

Concern over regulations will continue to rise for CAR-NK products with increasingly complex programmable circuits compared to single-edited products. Development of CAR-NK must prove all of the following: editing specifications, genome stability, stable clones, no replication-competent virus, predictable behavior of cytokines, and validation of safety switches. Additionally, regulatory authorities will also evaluate information on how immune evasion edits are regulated, expected duration of persistence, and long-term risk of secondary cancer for gene-altered allogeneic products (Zhang S. et al., 2023; Safarzadeh Kozani and Safarzadeh Kozani, 2025), As shown in Table 3 and Figure 2, different genome-editing targets can improve CAR-NK cell activity, safety, persistence, and tumor targeting, but edited cells must also pass genome-stability and safety testing before patient infusion. It is vital to keep in mind that merely increasing the number of genetic modifications is not sufficient to guarantee larger therapeutic benefits. Multiplex editing may provide gains in several functional characteristics at the same time but will probably also lead to a higher risk of unexpected genome changes and make the product more complicated for characterization. Therefore, the clinical value of genome-edited CAR-NK cells should be evaluated in terms of the gains in functionality, genomic stability, manufacturing complexity, and safety.

**TABLE 3:** Genome-editing targets that may improve CAR-NK cell function and safety.

Genome-editing target/module	Editing strategy	Added engineering value	Functional outcome for CAR-NK therapy	References
TGF-β/SMAD resistance module	SMAD4 disruption or TGF-β pathway blockade	Converts suppressive TGF-β resistance into an intrinsic NK-cell property	Maintains cytotoxicity, cytokine secretion, proliferation, and tumor infiltration under TGF-β/activin-rich suppressive niches	(Rea et al., 2025; Thangaraj et al., 2024)
CD33 fratricide-control module	CD33 deletion before CD33-directed CAR/iNK engineering	Addresses AML targeting while reducing self-recognition during product expansion	Supports CD33-directed AML killing and improves feasibility of manufacturing CD33-focused NK/iNK products	(Li et al., 2025a; Wang et al., 2025b)
CD38 self-antigen deletion	CD38 knockout combined with CD38-CAR expression	Separates therapeutic CD38 recognition from NK-cell self-targeting	Reduces CAR-mediated fratricide and supports CD38-directed activity against myeloma, lymphoma, and AML models	(Troy et al., 2024; Edri et al., 2023)
Hypoimmune allogeneic-editing module	HLA-ABC knockdown plus HLA-E/PD-L1 expression or CD54/CD58 ablation	Adds immune-evasion capacity without relying only on lymphodepletion	Reduces host T-cell and NK-cell rejection while preserving off-the-shelf CAR-NK/iNK antitumor function	(Hammer et al., 2024; Liu et al., 2025)
CRISPR-screen-derived resistance nodes	Functional editing of NK-cell and tumor-cell regulators identified by unbiased screens	Moves target selection beyond obvious checkpoints toward experimentally discovered resistance circuits	Identifies edit combinations that may improve NK metabolic fitness, cytokine output, immune-synapse formation, and repeat killing	(Biederstädt et al., 2025; Guia et al., 2025)
KLRC1/NKG2A with FAS co-editing	Dual inhibitory-receptor and survival-pathway editing	Extends checkpoint editing from stronger activation toward longer functional persistence	Improves resistance to HLA-E/NKG2A inhibition and supports longer cytotoxic capacity after repeated stimulation	(Mietz et al., 2026; Qin et al., 2024)
CXCR4 trafficking module	CXCR4 knock-in, cotransduction, or enforced expression	Adds anatomical programming to genome-edited CAR-NK design	Improves migration toward CXCL12-rich bone marrow niches and may strengthen BCMA-CAR-NK activity in multiple myeloma	(Moles et al., 2024; Yazdanparast et al., 2025)

**FIGURE 2:**
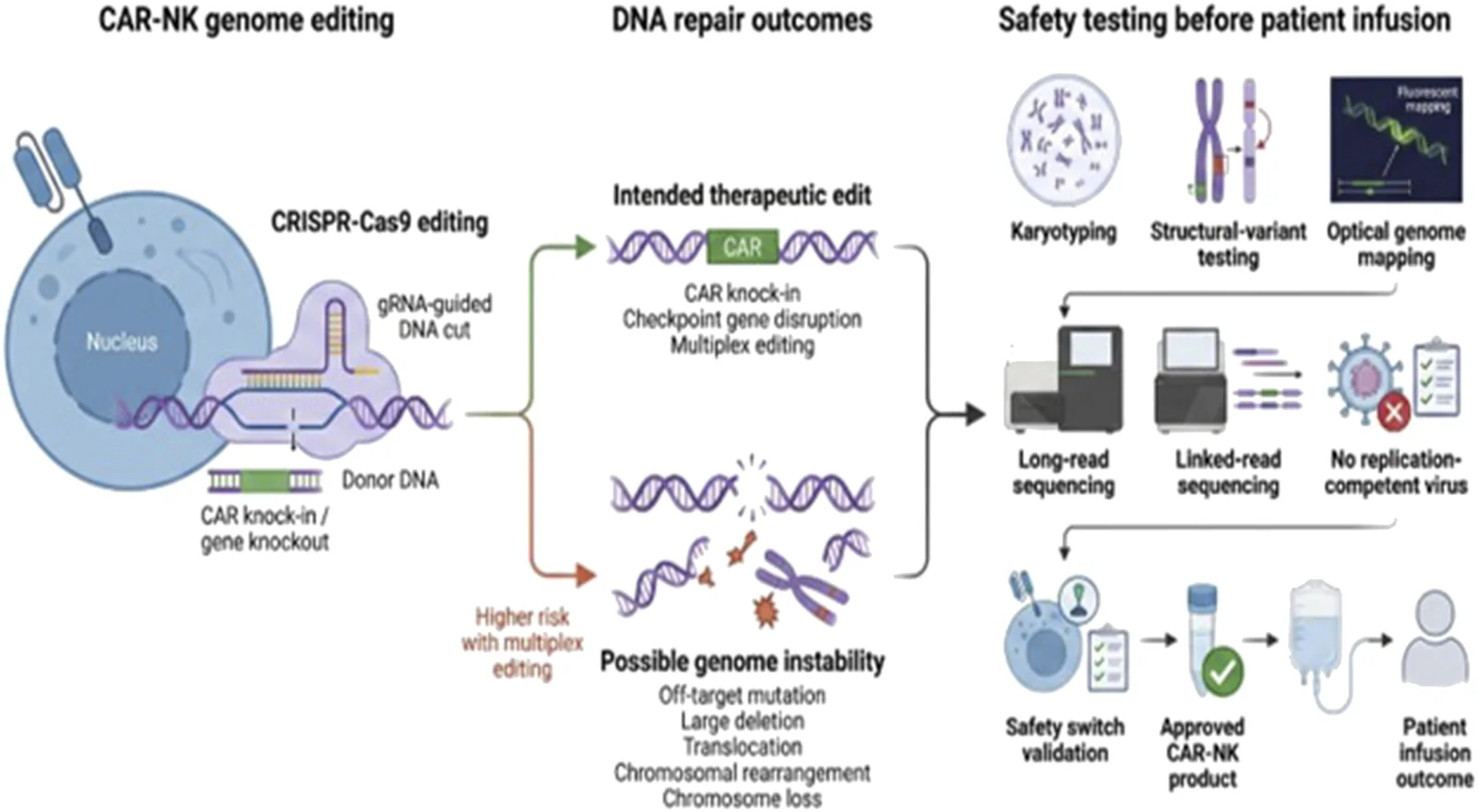
Genome editing and safety testing in CAR-NK cells. The figure summarizes intended editing outcomes, possible genome damage, and key safety checks before patient infusion.

## 4 Synthetic biology and programmable CAR-NK systems

The use of synthetic biology is changing CAR-NK therapy from an antigen-directed cytotoxic therapy into one that builds programmable designs of immune cells. It is possible to design artificial parameters that allow for sensing, signaling, cytokine release, trafficking, persistence, and termination as the functions of a modular therapeutic entity. This change in CAR-NK therapy will have a significant impact on hematological malignancies because relapses are affected by antigen plasticity, suppressive marrow environments, and heterogeneous immunity escape programs (Teng et al., 2024; Page et al., 2024).

### 4.1 Synthetic immunology principles

Instead of viewing immune cells as a fixed drug delivery system, in synthetic immunology, the immune cell is seen as an engineerable biological system or tool. In CAR-NK therapy, this allows the rational redesign of CAR-NK cells with regard to receptor specificity, namely, their ability to detect foreign substances; intracellular signaling; their dependence on cytokines for activity; the ability to resist inhibition; and persistent product performance after injection into the patient. Given that NK cells’ innate biological systems already utilize a combination of activating and inhibitory signals before committing to cytotoxic events, they are particularly amenable to this approach (McErlean and McCarthy, 2024; Hu et al., 2025).

The transition from non-programmable CARs, which are static or fixed cells such as CAR T cells, to programmed CAR natural killer cells represents an underlying mechanistic shift. Typically, conventional CARs are based on receiving the input from one main antigen; however, with synthetic cells it is possible to create or codify threshold sensing, conditional outputs, orthogonal signaling pathways, and context-dependent activation. These developments have particular significance in terms of the activation of NK cells against tumors originating from hematological malignancies, as both neoplastic and normal hematopoietic cells express shared lineage antigens (Biederstädt and Rezvani, 2025; Dos Reis et al., 2025).

The programmable CAR-NK design must also maintain translational feasibility, as complex circuitry may be ineffective if the original state of each cell type is preserved during expansion, cryopreserved, maintains its genomic stability, and maintains predictable potency post-thawing. Thus, developing a synthetic immunology approach to CAR-NK therapies requires careful consideration of both the complexity that is added to a design and the potential for engineered functions to remain viable for off-the-shelf manufacturing and clinical release testing (Jørgensen et al., 2025; Testa U. et al., 2025).

### 4.2 Logic-gated CAR-NK cells

The intent of creating a CAR-NK cell with Logic Gates is to provide a means to address the issue of specificity due to the presence of shared hematopoietic antigens. An AND gate logic system requires at least 2 inputs for full activation, thus reducing the chance of killing cells expressing only 1 of the 2 markers. This makes AND-gated logic CAR-NK an attractive solution for targeting AML (Acute Myeloid Leukemia) and myeloma, where tumor-associated antigens are rarely tumor-specific and there is a large amount of phenotypic diversity in the antigen density among subclones of each disease (Hamieh et al., 2023; Kondo et al., 2025).

The use of OR-gate systems will make it easier to find tumors since either of two separate antigens can lead to activation of the system, which will decrease the chance of relapse due to escape from antigen-negative cells. The incorporation of NOT-gate systems adds an additional level of inhibition to the CAR-NK by suppressing activation when a protective antigen is expressed on normal tissue. With these changes, the combined circuitry allows for a shift in the specificity of CAR-NK cells from single-marker recognition to combinatorial discrimination between malignant identities (Bangayan et al., 2023; Schindler-Wnek et al., 2025).

Multi-layer logic circuits have significant relevance for hematologic malignancies given that phenotypic overlap between leukemic blasts, leukemic stem cells, plasma cells, and normal progenitors exist. A programmable CAR-NK product can simultaneously integrate the presence of tumor antigens, the absence of healthy-cell antigens, and the expression of stress ligands prior to degranulation, namely the dumping of toxic granules. The logic-based architecture of such a product may allow improved selectivity while maintaining the recognition properties of innate NK cells without compromising them (Peng et al., 2024; Zugasti et al., 2025). As shown in Figure 3, CAR-NK cell engineering is transitioning from conventional receptor-based cytotoxicity to programmable, synthetic biology–driven systems capable of logic-gated recognition, inducible cytokine control, and autonomous decision-making within tumor microenvironments.

**FIGURE 3:**
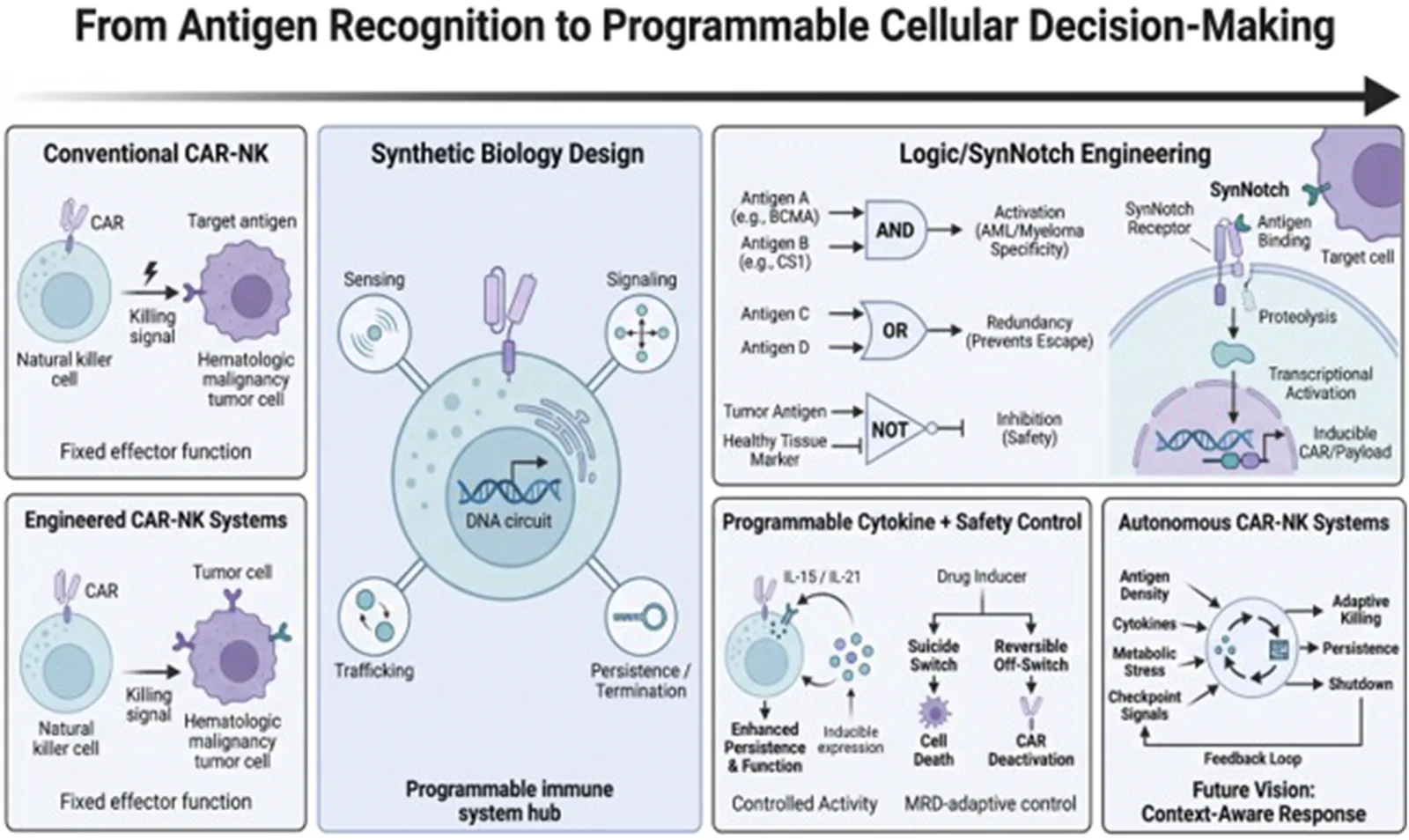
From antigen recognition to programmable cellular decision-making in CAR-NK cells.

### 4.3 SynNotch platforms

The architecture of SynNotch affords sequential recognition through the decoupling of sensing and executing. Antigens are directly bound to the CAR (chimeric antigen receptor), activating it; however, an antigen must first bind to a SynNotch receptor before a second therapeutic module is engineered to trigger transcription in response to an activation of the first antigen bound to the SynNotch receptor. This provides greater specificity with regard to CAR+ NK systems with respect to the density of CAR expression, levels of cytokine release, or the inhibition of checkpoint proteins within an appropriate contextual environment surrounding antigens (Lupo et al., 2024; Shirzadian et al., 2025).

Sequential recognition can allow an antigen to provide contextual information about a disease but cannot act as a direct target for lethal activity. A synNotch receptor can detect this contextual antigen and activate a CAR, cytokine, or other immune-modulating payload against a different antigen. This method of activating the cargo is both spatially and transcriptionally restricted, providing greater specificity than just expressing the CAR constitutively (Semeniuk et al., 2024; Wang T. et al., 2024).

The development of synNotch as part of CAR-NK would be based around minimizing basal leakiness while also having NK-cell effector kinetics that are rapid. Due to the fact that NKs kill faster than circuits for transcriptional control develop, the timing of the circuit should and will correspond with the biology of degranulation, the production of cytokines, and the duration of time the NK is in contact with its target. The use of improved receptor architectures may also allow for expansion of synNotch from sensing surface antigens into a much broader programmable receptor system (Piraner et al., 2025; Yu et al., 2025).

### 4.4 Programmable cytokine release

Cytokine release via genetic programming will be vital for successful CAR-NK optimization since NK cells generally demonstrate diminished persistence *in vivo* when compared to CAR-T cells. Specifically, IL-15 is critical since it promotes NK-cell survival, proliferation, and maturation to become cytotoxic and maintain metabolic fitness. Conversely, if there is a lack of control over the levels of IL-15 being produced or released, then excessive expansion of NK cells, or inflammation resulting from excessive activation of NK cells, would occur; therefore, controlled architectures of cytokines must be utilized (Baughan et al., 2025; Xu et al., 2025).

The purpose of using injectable IL-15 systems is to separate continuous exposure to cytokines from their long-term effects. Therefore, the production of cytokines should be high when there are interactions with tumors and decrease once the tumor has been destroyed. In addition, understanding how these pathways work at a mechanistic level is important as it relates to IL-15 activating STAT5 as well as driving through mTOR metabolic pathways while simultaneously inducing cytotoxic granule pathways and anti-apoptotic pathways; however, these pathways can also lead to toxicity if they remain activated long-term (Marr et al., 2025; Berjis et al., 2024).

Cytokines that are anchored to a membrane, as well as receptors that are capable of binding chimeric cytokines or combining these two types of cytokine activations into a single functional receptor by coupling, can be used to deliver localized supportive signals only to the engineered cells or tumor microenvironment. In hematological malignancy models, this approach may help to maintain the persistence of CAR-NK cells within the marrow and lymphoid compartments without having to expose an entire patient to a system-wide effect of cytokine pressure (Chen S. et al., 2024; Jiang et al., 2025).

A key objective of CAR-NK cell therapy for the treatment of cancers, especially leukemias and lymphomas, is to develop CAR-NK cells that do not require external sources of cytokines, such as IL-15, for continued proliferation and differentiation. Induction of IL-15 in CAR-NK cells through the use of an IL-15 knock-in or through regulated IL-15 expression, such as the use of a doxycycline-inducible promoter, will promote the survival, proliferation, fitness, and maturation of CAR-NK cells, thus enhancing their ability to perform immune surveillance of residual disease following cytoreductive therapy for leukemia or lymphoma (Wang X. et al., 2025; Luo et al., 2025).

Engineering IL-21 provides a supportive option to the similar goal of NK cell expansion, supporting more metabolic activity and NK cell function under specific conditions. The use of an autocrine cytokine circuit could lessen reliance on systemic use of cytokines but needs to be designed to eliminate any risk of excessive expansion. Triple configurations with binding from overall access to the tested cytokine, safe switch, and restricted expression to the tumor will likely be most effective (He et al., 2025; Dysthe et al., 2025).

### 4.5 Safety switches

Safety switches are critical to provide irreversible control of CAR-NK cellular therapy upon administration of a triggering drug to eliminate infused cells. Armored CAR-NK products have unique considerations for a safety switch due to increased biological activity from cytokine support, resistance to checkpoint inhibition, and edits that increase persistence, all of which can lead to greater risk for the patient (Lu et al., 2024; Cochran et al., 2025).

Controlled drug switches provide more versatility for controlling suicide systems versus those that are permanent in nature. Counsels can tune suicide cell product expression or assemble systems receptor assemblies and tune signal intensity in regard to the use of small residue-based, molecule-based, or compound-based drugs. In terms of CAR-NK therapy, reversible control will enable the physician to stop activity when there is a side effect from the medication and restore activity again if there is no residual disease left (Liu Y. et al., 2024; Giordano Attianese et al., 2024).

Extracellular reversible activation mechanisms are conceptually useful for precision immunotherapy since the hematologic tumor load can change rapidly after therapy. If the patient has achieved deep remission, it may not require a continuous active cell product. In contrast, the continued use of a continuously active cell product may create a risk for the patient if there is inflammation. Adjustable CAR expression and switches that are regulated by small molecules could help to harmonize CAR-NK functionality with tumor kinetics, toxicity, and the status of measurable residual disease (MRD) (Zajc et al., 2024; Głowacki et al., 2024).

Synthetic biology approaches may provide additional strategies to control immune-related toxicities by incorporating regulatory circuits that limit excessive immune activation. Examples include inducible safety switches, such as suicide gene systems, which allow selective elimination of engineered cells in the event of severe toxicity, as well as synthetic logic-gated circuits that restrict CAR activation to specific tumor-associated signals. However, most of these safety mechanisms have been developed and experimentally validated primarily in CAR-T cell platforms, whereas their application in CAR-NK cells remains comparatively limited and is still undergoing preclinical evaluation. Therefore, further studies are required to optimize and validate synthetic regulatory systems specifically for CAR-NK therapy (Lu et al., 2024; Dehghan et al., 2025).

### 4.6 Autonomous cellular therapeutics

Autonomous CAR-NK therapies exemplify the highest goal in synthetic immunology. Rather than recognizing just one antigen, these systems would have the capacity to detect stimulation from various environmental cues and respond with an appropriate output based on the context through the processing of these multiple signals. The connection between molecular stimuli to create synthetic gene circuits will be the foundation for developing such cellular therapeutics using closed-loop systems (Piraner et al., 2025; Głowacki et al., 2024).

Autonomous CAR-NK cells may use antigen density, suppressive cytokines, checkpoint ligands, metabolic stress, and chemokine gradients to integrate their information prior to making a decision about killing, persistence, trafficking, cytokine secretion, or becoming inactive in the context of hematologic malignancies. Indicators of resistance that will inform the creation of these adaptive engineering programs may be identified through genome-wide and *in vivo* CRISPR screening (Rea et al., 2025; Peng et al., 2025).

Autonomous CAR-NK treatment has clinical value based on predictable variables rather than on maximum complexity. The closed-loop circuits must demonstrate low basal activity, stable genomic integration, controllable expansion, and compatibility with the mechanisms that allow for allogeneic immune evasion. The end result is an adaptable cell therapy product that senses malignant ecology, adapts to resistance to therapy, and self-limits when therapeutic pressure is no longer required (Biederstädt and Rezvani, 2025; Martin et al., 2024), as shown in Table 4. Synthetic biology tools support the design of programmable CAR-NK cells by improving control, persistence, safety, and tumor-specific activity.

**TABLE 4:** Synthetic biology tools in CAR-NK engineering.

Synthetic biology tool	Added engineering value	Functional contribution to CAR-NK design	Translational checkpoint	Refrences
NK-tuned CAR signaling domains	Uses signaling modules that fit NK-cell biology rather than copying CAR-T designs	Improve serial killing, persistence, and post-thaw potency	Test tonic signaling, degranulation, cytokine release, and exhaustion after expansion	(Acharya et al., 2024; Yi et al., 2025)
Multi-input logic circuits	Combines activating and protective antigen signals in one product	Help target AML or myeloma when tumor antigens overlap with normal blood cells	Confirm receptor balance and low killing of healthy antigen-positive cells	(Clubb et al., 2023; Frankel et al., 2024)
SynNotch payload control	Links antigen sensing to local expression of a CAR, cytokine, or blocking payload	Convert tumor contact into a restricted therapeutic output instead of constant activation	Measure basal leakiness, response time, and payload diffusion	(Lupo et al., 2024; Cortese et al., 2024)
Cytokine fitness modules	Treats IL-15 or IL-21 as a controlled survival program, not simply an activation booster	Support CAR-NK persistence in marrow and lymphoid niches without relying only on systemic cytokines	Link cytokine level to expansion, toxicity, and safety-switch performance	(Marin et al., 2024; Van Den Eynde et al., 2024)
Multiplex precision editing	Combines CAR expression with edits that remove intracellular brakes or inhibitory checkpoints	Help engineered NK cells resist suppression and repeated tumor rechallenge	Requires off-target, karyotype, persistence, and exhaustion testing	(Wang et al., 2025a; Rafei et al., 2025)
External ON/OFF control	Allows drug- or adaptor-dependent tuning of CAR activity after infusion	Could CAR-NK activity match tumor burden or MRD status instead of keeping constant pressure	Needs NK-specific validation of reversibility, dosing, and immunogenicity	(Kvorjak et al., 2023; Pfeifer Serrahima et al., 2023)

## 5 Engineering persistence and overcoming resistance

### 5.1 Biological basis of CAR-NK failure

Although CAR-NK cells have demonstrated a favorable safety profile, their limited long-term persistence remains a major challenge for achieving durable therapeutic responses, particularly in solid tumors. Compared with CAR-T cells, conventional NK cells generally exhibit shorter *in vivo* persistence and limited proliferative expansion after infusion. In addition, the absence of continuous homeostatic cytokine support, including IL-15 signaling, may restrict NK-cell survival and expansion after transfer into the recipient. Consequently, CAR-NK engineering strategies are increasingly focused on improving persistence while maintaining controlled activation and minimizing potential toxicity (Xie et al., 2020).

A third important limitation is functional exhaustion. The persistent exposure to antigens in the tumor microenvironment leads to progressive functional exhaustion of the T cell transcriptional and epigenetic programming, reflected by a marked reduction in cytokine secretion, as well as a general inability of T cells to degranulate and express CD8^+^ cytotoxic activity, less cellular proliferative potential, and increased expression of inhibitory receptors, e.g., TIGIT, TIM-3, PD-1. In addition, constant inflammatory signaling combined with accumulation of metabolic stress produces mitochondrial dysfunction that limits ATP production and therefore cytotoxic efficacy in T cells. The exhausted phenotypes that are developed in T cells correlate directly with metabolic reprogramming, establishing both persistence of T cells and their overall fitness as important therapeutic targets for future generations of CAR-NK engineering (Dias et al., 2024).

Therapeutic efficacy of CAR-NK products is limited by host-mediated immune rejection, especially with regard to allogeneic “off-the-shelf” products. Allogeneic NK cells are inherently at lower risk of graft-versus-host disease than allogeneic CAR T cells; however, recipient T cells, macrophages, and remaining immune surveillance mechanisms recognize the infused NK cells as foreign and limit the duration of their persistence (Schmidt et al., 2022).

### 5.2 Metabolic engineering

Metabolic reprogramming is becoming an essential approach for enhancing the continued presence of CAR-NK in the tumor microenvironment (TME), which is a very hostile place. Activated NK cells demonstrate an immediate increase in both glycolysis and mitochondrial oxidative phosphorylation (OXPHOS) to support the release of cytotoxic granules, as well as cytokine secretion and proliferation. However, chronic antigen stimulation in addition to nutrient deprivation led to mitochondrial dysfunction as well as decreased ATP production, increased reactive oxygen species (ROS) production, and a gradual reduction of effector function. Therefore, engineering strategies are directed toward preserving the integrity of the mitochondria through enhancement of mitochondrial biogenesis, maintenance of mitochondrial membrane potential, and optimization of mitochondrial respiratory capacity to maintain long-term cytotoxicity (Dehghan et al., 2025).

Concurrently, engineering ways to resist oxidative stress represent another important design challenge. Excessive oxidative stress (ROS) generated within hypoxic, low-oxygen tumors causes damage to mitochondrial DNA, proteins, and lipid membranes, leading to an accelerated rate of functional decline. By overexpressing antioxidant regulatory proteins, enhancing glutathione metabolism, and altering mitochondrial quality control pathways, it has been shown that NK-cell viability can be preserved in an oxidative environment. In addition, these strategies maintain cytokine chemical signaling intermediates production while delaying metabolic dysregulation associated with cellular exhaustion (McErlean and McCarthy, 2024; Dehghan et al., 2025).

One of the biggest challenges for developing CAR-NK therapies is that both immune cells and tumors need to access nutrient resources such as glucose, amino acids, and lipids. Since tumors quickly use up these nutrients, CAR-NK therapy can affect immune function because CAR-NK cells cannot gather the same nutrition as tumors do. Therefore, current engineering strategies for CAR-NK therapies are focused on allowing CAR-NK cells to obtain more glucose by increasing their metabolic flexibility through fatty acid oxidation and by taking up more nutrients, glucose, amino acids, and lipids, and optimizing mTOR-dependent signals controlling cell metabolism, thus allowing engineered NK cells to keep functioning as cytotoxic agents in low-glucose tumor microenvironments (Schmidt et al., 2022).

### 5.3 Realistic prospects for fixing the major limitations of each platform

#### 5.3.1 CAR-T cells

Graft-versus-host disease is the biggest challenge for developing ready-to-use allogeneic CAR-T therapies. This occurs because donor T cells carry their own functional T-cell receptors. However, scientists have successfully overcome this by knocking out the TCRα gene (TRAC), a strategy that has been validated in several clinical products, making this a solvable problem in the near future (Balkhi et al., 2025). In parallel, base-edited multiplex allogeneic CAR-T manufacturing has demonstrated lower genotoxicity and higher yields than Cas9-nuclease editing, suggesting that safer, scalable allogeneic CAR-T products are achievable in the near to mid-term (Engel et al., 2025).

In contrast, cytokine release syndrome (CRS) and neurotoxicity present greater challenges for complete elimination due to their intrinsic mechanistic association with potent CAR-T cell activation. Nevertheless, risk-mitigation strategies including cytokine pathway blockade, inducible safety switches, and logic-gated or in vivo-generated CAR-T approaches are progressively reducing clinical severity, although achieving a definitive solution remains an active area of investigation (Khanal et al., 2025).

#### 5.3.2 CAR-NK cells

Limited *in vivo* persistence is currently being addressed through cytokine-armoring strategies, with IL-15 representing the most prominent approach. In a phase 1/2 clinical trial evaluating IL-15-armored CD19 CAR-NK cells in patients with relapsed/refractory lymphoid malignancies, complete remission was achieved in 7 out of 11 patients, with cellular persistence documented for up to 1 year following infusion. These findings demonstrate that this strategy is already producing clinically meaningful improvements beyond preclinical observations (Baughan et al., 2025). This approach carries risks: while constitutive IL-15 secretion enhances CAR-NK cell persistence, it has triggered fatal systemic inflammation in some peripheral blood–derived products. Consequently, cytokine armoring must be carefully optimized according to the cell source and delivery method favoring membrane-bound over secreted IL-15 to ensure safety. This refinement remains an active yet solvable engineering challenge, not an insurmountable obstacle (Amarasinghe et al., 2026).

Manufacturing scalability is advancing as well: feeder-cell-free CAR-NK production methods that pharmacologically activate PKC and calcium-signaling pathways can generate high-quality CAR-NK cells with lower inflammatory potential. This eliminates the long-standing reliance on tumor-derived feeder cell lines, along with their associated safety and regulatory challenges (Khanal et al., 2025). Immune rejection of allogeneic CAR-NK products and residual heterogeneity in iPSC-derived sources remain more open challenges, but multiplex gene-editing strategies (e.g., B2M knockout, HLA-E overexpression, CD47 overexpression) combined with emerging AI-assisted construct design are being developed specifically to address these barriers, representing a realistic, if still maturing, path forward (Baughan et al., 2025). Overall, we characterize the CAR-T toxicity problem (CRS/ICANS) as partially mitigable but mechanistically persistent, the CAR-T GvHD problem as close to solved at the engineering level, the CAR-NK persistence problem as showing clinically validated early solutions with ongoing safety optimization, and the CAR-NK manufacturing/immune-evasion problem as promising but still in active development. This graded, evidence-based appraisal has been incorporated into the revised manuscript to give readers a realistic, rather than purely aspirational, view of near-term clinical translation.

## 6 Precision targeting and antigen escape prevention

The issue of antigen escape from CAR-NK cell therapy can now be viewed as a precision targeting challenge that is different from just selecting the target. Within hematopoietic cancers, tumor clones may be able to modify their antigen density, change their lineage identity, or change their surface phenotype when under attack from the immune system. Next-generation CAR-NK cell systems, therefore, will require an architecture that has both NK cell intrinsic recognition combined with multiple antigen targeting and modular/retargetable receptors (Zhong and Liu, 2024; Bastrich et al., 2025).

### 6.1 Mechanisms of antigen escape

Antigen escape occurs when CAR pressure selectively eliminates antigen-high cells while antigen-low or antigen-negative clones survive; this could reflect downregulation of the antigen itself, alterations in exon levels due to splicing, loss of epitope, or expansion of pre-existing populations of CD19-negative cells that were already CD19-negative. Native activating receptors may partially compensate for this; however, CAR specificity would continue to remain subject to the dominant loss of antigen (Mishra et al., 2024; Nasiri et al., 2025).

Antigen escape due to clonal evolution is particularly problematic in leukemia, lymphoma, and myeloma because relapsed malignancies do not represent a static recurrence of original tumors. Treatment can alter malignant populations by selecting for subclones with lower levels of target antigen density, a different degree of differentiation, or potential for lineage switching, thus necessitating both pre-treatment antigen mapping and post-treatment follow-up prior to making a decision to use a single-antigen CAR-NK product (Bartoszewska et al., 2024; Liu Z. et al., 2024).

In addition to antigen recognition and granzyme-mediated cytotoxicity, CAR-NK cells can induce tumor cell apoptosis through death receptor pathways, including the Fas (CD95)/Fas ligand (FasL) axis. Alterations in Fas expression or impaired sensitivity to Fas-mediated apoptosis may provide an additional mechanism of tumor escape by allowing antigen-low or antigen-negative clones to survive despite immune pressure. Consequently, selective expansion of Fas-resistant tumor populations may contribute to disease relapse following CAR-NK therapy. Understanding the interaction between antigen expression and apoptotic susceptibility is therefore important for developing strategies that overcome heterogeneous tumor resistance (Nguyen et al., 2024; Lin M. J. et al., 2025).

### 6.2 Dual and multi-target CAR systems

Dual-target CAR systems can reduce escape by requiring malignant cells to lose an additional relevant antigen before becoming invisible. In terms of structure, tandem CARs contain two binding domains in one receptor construct, while bicistronic vectors express two CAR constructs from a single vector. The clinical support for these two approaches is through their use in CD19/CD22 and CD19/CD20 hematological platforms and creates a potential design for CAR-NK translation (Roddie et al., 2023; Wang L. et al., 2024).

The bicistronic and co-transduced CD19/CD22 systems provide valuable information regarding the release mechanism of incomplete CD19 coverage seen in B-ALL. This same concept can be utilized in CAR-NK design for the treatment of CD19/CD22 lymphoma, BCMA/GPRC5D myeloma, or CD33/CLL-1 AML. The reoccurring issue surrounding such systems is finding a balance between expanded recognition of the target versus receptor competition, signaling imbalance, and size limitations of the vector (Ghorashian et al., 2024; Liu et al., 2023).

The theory behind trivalent designs expands the thought process by targeting three disease-related antigens or combining CAR recognition with NK-cell accessory functions like CD16-mediated ADCC. This is an attractive feature of CAR-NK therapy, because NK cells can integrate CAR signaling with natural cytotoxicity and ADCC. The design of the multi-target approach should be to create a coordinated immune response, rather than just stacking antigens on top of each other (Yang J. et al., 2024; Guo et al., 2024).

### 6.3 Universal CAR platforms

The anti-cancer agents of CAR are designed to identify and bind to multiple target antigens, rather than just one that will trigger the immune response. The flexibility of CAR allows one tract of cells containing NK cells to have access to an unlimited number of NK cell products by coupling NK cells with various antibodies or ligands for targeting CD19, CD22, CD33, BCMA, CD38, or CD70 in response to cancer types and phenotypes (Schlegel et al., 2024; Mohammad et al., 2024).

Adapters for CARs and SUPRA-type systems offer great value as the dose of the adaptor can theoretically modify the intensity of activation, the duration of the signal and the toxicity of the SIGNAL. For example, in patients with hematologic malignancies, this might allow for the switching of antigens following relapse without having to produce a new cell-based product. In CAR-NK cells, the off-the-shelf nature of the platform provides an opportunity for modularity, assuming that the pharmacokinetics of each adaptor are well understood (Park et al., 2024; Huang et al., 2025a). As shown in Figure 4, antigen escape in CAR-NK therapy arises through multiple evolutionary and phenotypic mechanisms, necessitating integrated engineering approaches including multi-target CAR design, universal adaptor systems, and dynamic retargeting strategies to sustain antitumor efficacy in hematologic malignancies.

**FIGURE 4:**
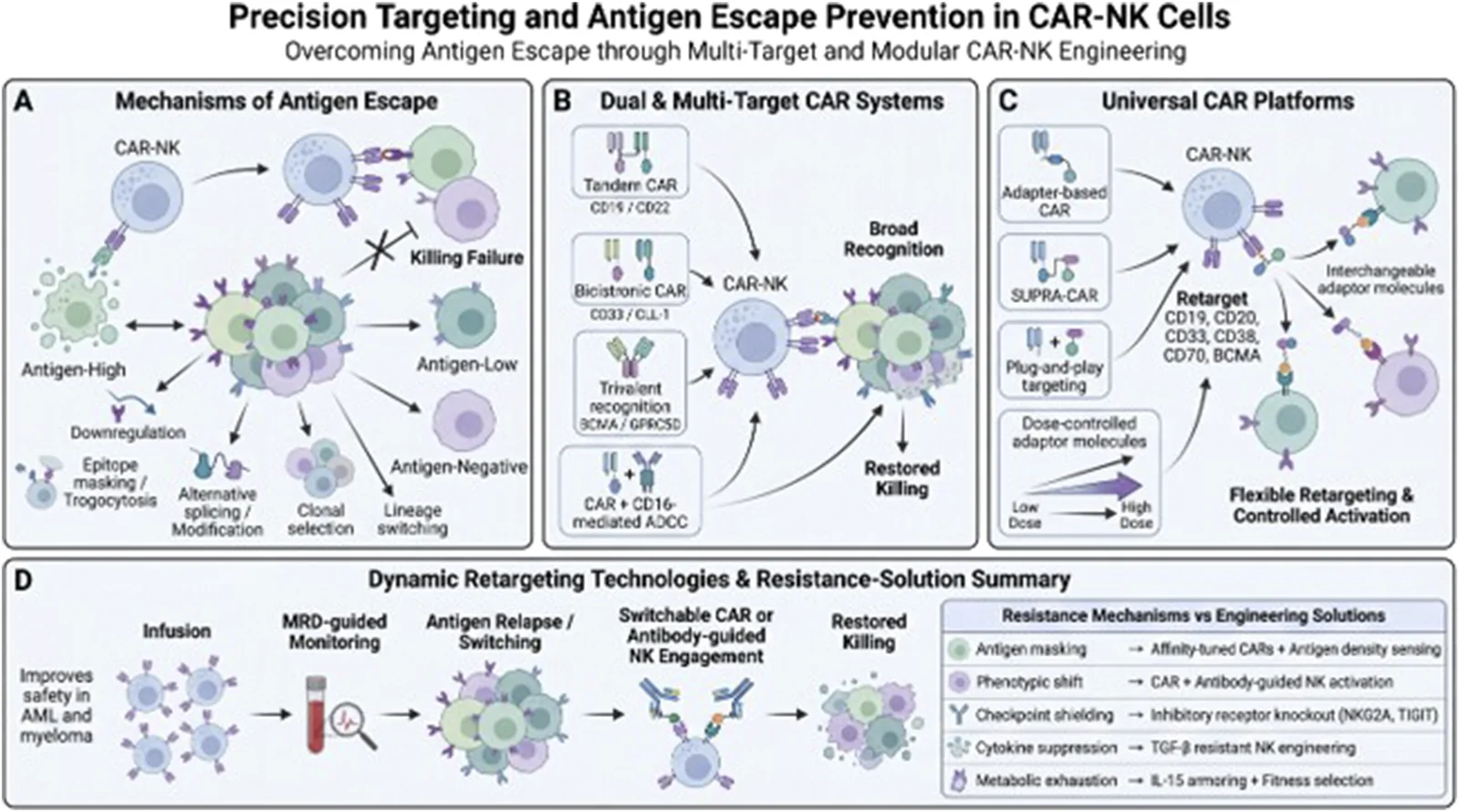
Precision Targeting and Antigen Escape Prevention in CAR-NK Cells. **(A)** Mechanisms of antigen escape, including antigen downregulation, epitope masking/trogocytosis, alternative splicing or modification, clonal selection, and lineage switching, leading to CAR-NK killing failure. **(B)** Dual- and multi-target CAR systems, including tandem CARs, bicistronic CARs, trivalent recognition, and CAR + CD16-mediated antibody-dependent cellular cytotoxicity (ADCC), enabling broader antigen recognition and restored killing. **(C)** Universal CAR platforms, including adapter-based CARs, SUPRA-CARs, plug-and-play targeting, and dose-controlled adapter molecules, enabling flexible retargeting and combinatorial activation. **(D)** Dynamic retargeting technologies and resistance-solution strategies, including MRD-guided monitoring, antigen relapse/switching, switchable or antibody-guided CAR-NK engagement, and restored killing. Figure 4 also summarizes resistance mechanisms and corresponding engineering solutions, including antigen masking, phenotypic shift, checkpoint shielding, cytokine suppression, and metabolic exhaustion.

### 6.4 Dynamic retargeting technologies

Dynamic retargeting technologies go beyond fixed antigen programming by allowing the ability to change recognition after infusion. Switchable CAR systems, antibody-guided NK engagement, and CAR-like NK platforms all provide the ability to change the cytotoxicity directed toward a tumor as its phenotype is shifting. This is especially pertinent in the context of CD19-negative relapse or BCMA-low progressive myeloma, where a static CAR-NK product may have become biologically irrelevant (Coënon et al., 2024; Zu et al., 2024).

Modular recognition systems may help with patient safety by allowing the temporary removal of any specific targeting adaptor if a toxic effect is noted. In the case of acute myeloid leukemia and the various ways that CD33, CD123, CLL-1, and FLT3 overlap with normal hematopoiesis, reversible retargeting of an effector system is a much more rational approach than applying a single antigen permanently. An ideal CAR-NK effector system would be able to adapt its target to the evolving level of measurable residual disease (Huang et al., 2025b; Abbaszade Dibavar et al., 2024), as demonstrated in Table 5. Resistance to CAR-NK therapy may arise from antigen loss, inhibitory signals, or weak cell persistence, and engineering strategies can help overcome these barriers.

**TABLE 5:** Resistance mechanisms and engineering solutions.

Resistance pressure	Engineering solution	Design value for CAR-NK therapy	References
Trogocytosis-driven antigen masking	Affinity-tuned CARs with post-infusion antigen-density monitoring	Reduces transient target stripping and limits CAR-NK dysfunction after early tumor contact	(Chen et al., 2024b; Ramezani et al., 2023)
Phenotypic shift after single-antigen pressure	CAR plus Fc-receptor or antibody-guided recognition layer	Keeps one NK product redirectable when relapse changes the dominant antigen profile	(Ghobadi et al., 2025; Meng et al., 2023)
Inhibitory checkpoint shielding	NKG2A-edited or inhibitory-axis-aware CAR-NK design	Preserves killing when tumor cells retain the target antigen but increase NK inhibitory signals	(Bexte et al., 2024a; Schindler-Wnek et al., 2025)
Suppressive cytokine imprinting	TGFβ/activin-resistant NK programming	Maintains cytotoxicity, cytokine secretion, and proliferation within suppressive marrow or lymphoid niches	(Rea et al., 2025; Kong et al., 2024)
Post-response metabolic exhaustion	IL-15/IL-15RF armoring with product-fitness release criteria	Treats persistence and metabolic fitness as anti-resistance endpoints, not only potency enhancers	(Li et al., 2023a; Marin et al., 2024)

## 7 Multiomics and AI-guided CAR-NK engineering

Through multiomics and AI-guided engineering, the development of CAR-NKs has shifted from an empirical process of receptor selection to a data-driven paradigm of cellular design. Rather than selecting receptors based solely on bulk antigen expression, we now use an integrated approach that combines information from a variety of single-cell, spatial, proteomic, and computational models to inform receptor selection and CAR architecture design. These models include assessing malignant identity, immune resistance profiles, niche localization, and patient-specific vulnerability. Taken together, these models will support our efforts to engineer CARs for optimal receptor selection and trafficking strategies (Le et al., 2025; Luciani et al., 2025).

### 7.1 Single-cell multiomics

Single-cell RNA sequencing makes it possible to use functional cell states rather than average levels of tumor expression for the start of CAR-NK engineering. For AML, single-cell transcriptomics have provided evidence of dysfunctional, inflammatory, and exhausted CD3^+^ T and NK cells with respect to these cells’ effector function and has also demonstrated the presence of blast-associated immune-suppressive programs that may also serve to suppress cytotoxic cells. These data will help identify CAR-NK failure and if it is caused by loss of antigens, immunologic exclusion, inhibitory signaling, or impaired effector cell fitness (Zhang Z. et al., 2023; Hu et al., 2024).

scATAC-seq provides an additional level of information by determining the chromatin programs that govern lineage flexibility, resistance to therapy, and evasion of the immune system. When designing CAR-NK products from transcriptionally related malignant cells, for example, those with definition and stemness regulatory characteristics may also differ at the level of enhancer accessibility or have been primed for stress response. Using the profiles of the malignant cells can also assist in the identification of genome edits that would maintain the cytotoxic programs of these cells and help to overcome any epigenetic states that would confer resistance to these therapies (Fan et al., 2023; Li X.-P. et al., 2025).

CITE-seq, in conjunction with single-cell proteogenomics, is beneficial for selecting CAR-NK targets since mRNA levels do not always correlate with surface antigen density. In multiple myeloma, single-cell immune mapping can provide valuable information regarding malignant plasma cell heterogeneity, immune cell suppression, and surface marker variability throughout different stages of precursor and advanced disease, helping support the rational selection of CAR-NK-based strategies directed at BCMA, GPRC5D, CD38, or SLAMF7 (Pilcher et al., 2023; Cheng et al., 2024).

### 7.2 Spatial biology

Spatial transcriptomics enhances the anatomical representation of multiomics to reveal how malignant cells interact with immune cells within the bone marrow and lymphoid tissues. In patients with AML, spatial profiling can demonstrate how therapies can cause stromal, myeloid, and immune neighborhoods to change physically to restrict CAR-NK access. Additionally, these spatial maps will guide the engineering of chemokine receptors and ways to adapt armor against these microenvironments (Gui et al., 2025; Cooper et al., 2025).

Spatial biology plays an important role in understanding multiple myeloma, in particular the fact that malignant plasma cells exist in specialized bone marrow locations that are created by surrounding stromal cells, osteolineage cells, blood vessels, and immunosuppressive populations. By spatially profiling transcriptomic data from trephine biopsies, it will be possible to determine if CAR-NK cell therapy requires improved homing to the bone marrow, improved stromal penetration, or additional localized cytokine support to access compartments that protect from disease (Yip et al., 2025; Muiños-Lopez et al., 2025).

By utilizing spatial proteomics and mapping of cellular neighborhoods, one can find receptor-ligand interactions that could not otherwise be detected in separate single-cell datasets. In the setting of lymphoma or bone marrow biopsies, the above technologies may yield an understanding of how specifically tumor cell survival is affected by either macrophage-rich, T cell-excluded, or checkpoint-dense niches. This knowledge will help guide the development of therapies for CAR-NK-resistant tumors via local delivery of cytokines or selection of combination therapies (Younes et al., 2024; Yeung et al., 2025).

### 7.3 AI-driven target discovery

Target discovery enabled by AI can integrate single-cell expression, tissue health restrictions, antigen density, and clonal prevalence to identify safer and more effective targets for hematological diseases. In AML, atlas-driven approaches already demonstrate how surfaceome and transcriptome datasets can be used to identify candidate antigen combinations as opposed to conventional single marker approaches, thereby supporting a more selective design of CARs (Gottschlich et al., 2023; Bordeleau et al., 2024).

Antigen selection should be thought of as a prediction, instead of just an exercise in listing markers. Algorithms can rank the target’s malignant coverage, the normal hematopoietic overlap, co-expression of antigens, the stability of relapses, and the compatibility with logic-gated recognition for the best antigen selection. This information is particularly applicable to AML and a new avenue for targeting combinations of antigens and future novel intracellular-derived surface targets in separating leukemic identity and normal progenitor biology (Atar et al., 2024; George et al., 2026).

Predictions of resistance are just as important because CAR-NK failure due to low antigen density, suppressive inflammation, ineffective trafficking, or dysfunction of the effector can occur. There are existing examples of prediction technologies used as a basis for determining response or toxicity, from using imaging, inflammatory signals, and clinical variables to make predictions for CAR-T treated lymphoma. These same predictive models can be utilized to develop new technologies for armoring CAR-NK therapy, checkpoint editing and patient selection for CAR-NK therapy (Tong et al., 2023; Raj et al., 2025). As shown in Figure 5, multiomic technologies, spatial biology, artificial intelligence, and digital twin modeling converge to form an integrated CAR-NK engineering pipeline that enables patient-specific target selection, predictive therapeutic modeling, and iterative optimization of engineered cellular therapies in hematologic malignancies.

**FIGURE 5:**
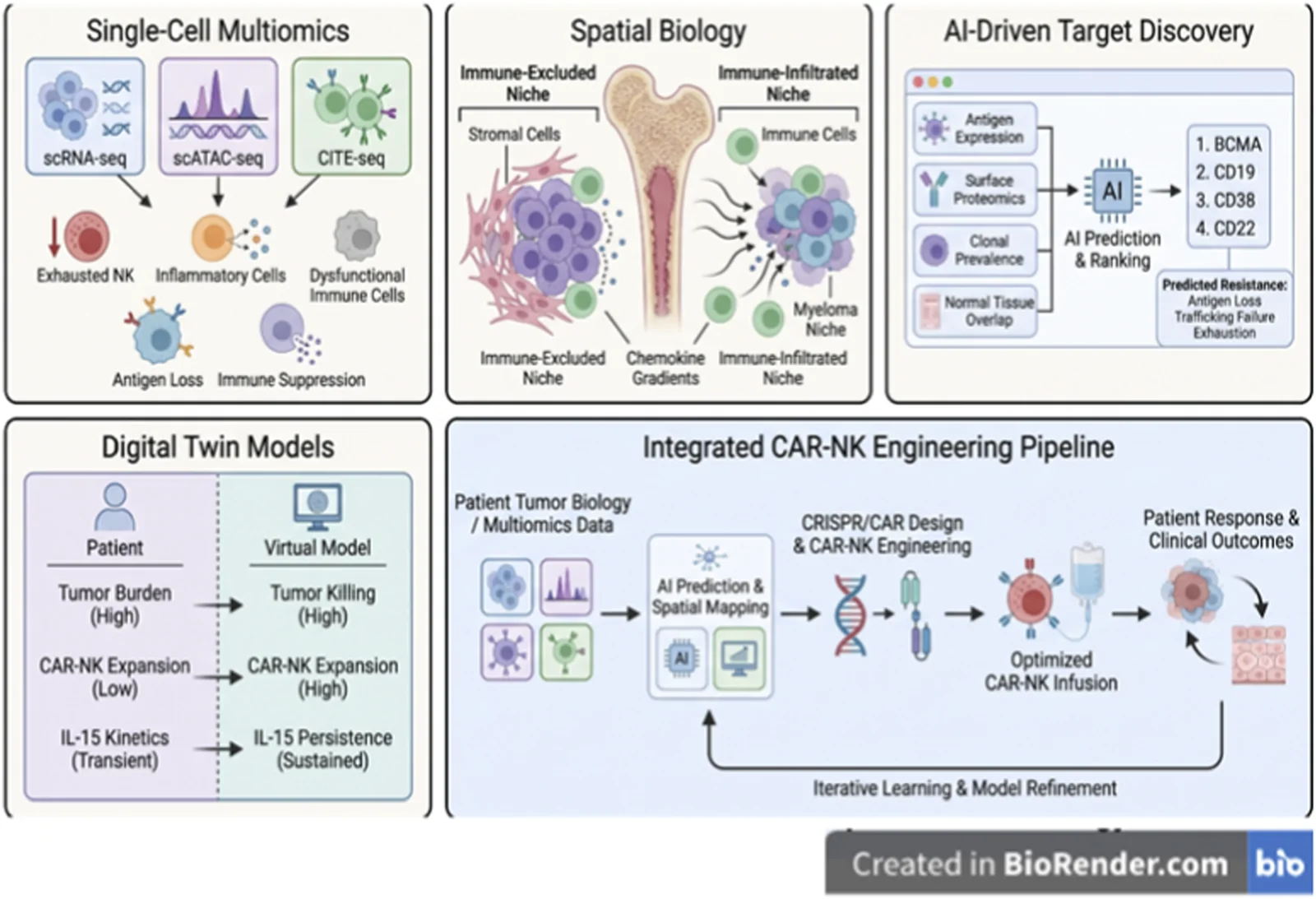
Multiomics and AI-guided CAR-NK engineering for precision immunotherapy.

### 7.4 Digital twin models

. A digital twin is a dynamic, computationally maintained virtual representation of a physical system in this context, a patient, a tumor, or an engineered cell product that is continuously updated with real-world data so that simulated behavior tracks the actual physical counterpart over time (Clement David-Olawade et al., 2026). As it is useful to distinguish three levels of digital representation that are often conflated: a digital model is a standalone virtual representation built from data, rules, or simulations but with no live connection to the physical system it represents; a digital shadow adds a one-way data link, so that information from the physical system automatically updates the virtual model in real time, enabling monitoring but not intervention; a true digital twin closes the loop with a two-way connection, in which the virtual system not only receives data from, but can also inform or influence, the physical system, for example, by recommending a dose adjustment or a manufacturing change that is then implemented in the real cell product or patient (Aghamiri and Amin, 2025).

In CAR-T and, by extension, CAR-NK cell therapy, digital twins have been proposed as a way to provide a virtual representation of the therapy–tumor trajectory using high-dimensional patient data, integrating parameters such as CAR-cell expansion kinetics, tumor burden, cytokine dynamics, and the immunosuppressive tumor microenvironment, in order to predict individual treatment trajectories and support real-time therapeutic adaptation (Zhang et al., 2025). Concrete applications already explored include patient-specific digital twins built to anticipate cytokine release syndrome after CAR-T infusion, so that prophylactic or early intervention (e.g., tocilizumab administration) can be planned before overt clinical decompensation occurs, illustrating how the concept moves from an abstract modeling framework toward a bedside decision-support tool (Aghamiri and Amin, 2025).

Extending this framework to CAR-NK cell therapy, patient-specific digital twin models incorporating tumor burden, tumor microenvironment, immune inhibition, cytokine kinetics, and cellular expansion can augment AI-guided engineering activities during therapy design. Prior to manufacturing, such models can help estimate whether a given patient will require increased persistence, dual targeting, marrow-homing enhancement, or reduced cytokine output (Politi, 2020; Mueller et al., 2025). Because CAR-NK efficacy depends on dynamic, dose-dependent interactions—including tumor killing, expansion, cytokine feedback, and immune evasion—these mechanistic models can simulate virtual doses and persistence before a product enters clinical trials, and can further assess IL-15-armored, safety-switch-threshold, and exhaustion-resistant CAR-NK designs (Luo et al., 2026; Baughan et al., 2025).

Rather than providing overly confident or “perfect” predictions, digital twins are best used to outline and test assumptions about these variables before treatment. Used this way, an advanced CAR-NK digital twin can predict and compare potential treatment outcomes for a given patient by weighing receptor design, genomic background, armoring strategies, and engineering approaches—including risks such as niche exclusion and loss of persistence—against alternative options at the virtual level, as summarized in Table 6 (Olawade et al., 2026).

**TABLE 6:** Emerging multiomic and AI approaches in CAR-NK development.

Approach	Main input	CAR-NK design decision	Added value	References
Surface proteogenomic filtering	scRNA-seq, CITE-seq, surface proteomics	Select antigens with real surface-level support	Avoids transcript-only target choices	(Knorr et al., 2023; Ramberger et al., 2024)
Spatial niche mapping	Bone marrow spatial transcriptomics and proteomic imaging	Guide homing or local armoring modules	Links CAR-NK design to tissue access	(Bandyopadhyay et al., 2024; Ly et al., 2025)
Functional CRISPR screening	NK-cell and tumor-cell perturbation screens	Prioritize edits that improve killing or resistance control	Moves editing from prediction to functional validation	(Biederstädt et al., 2025; Dufva et al., 2023)
Computational target ranking	Tumor/normal atlases and single-cell datasets	Rank safer single or paired targets	Reduces off-tumor risk before wet-lab testing	(He et al., 2024; Testa et al., 2025b)
Model-informed simulation	PK/PD, cellular kinetics, virtual-patient models	Compare dose, persistence, and cytokine-risk scenarios	Tests design assumptions before clinical translation	(Kirouac et al., 2024; Yang et al., 2025)

Beyond individual patient management, “virtual twin” frameworks have also been proposed at the product-design level for engineered adoptive cellular immunotherapies more broadly, supporting applications such as patient stratification, cost-effectiveness analysis for reimbursement models, and long-term safety monitoring (e.g., surveillance for rare insertional-mutagenesis-related secondary malignancies), positioning digital twins as a tool for both individualized dosing and systems-level manufacturing and pharmacovigilance decisions (Bastrich et al., 2025). Complementing this, multiomic and AI-based approaches can support CAR-NK development by improving target selection, tissue-homing design, functional editing, and model-informed translation.

### 7.5 Ribosome profiling (ribo-seq) as an emerging strategy for neoantigen and target discovery in CAR-NK engineering

Current chimeric antigen receptor natural killer (CAR-NK) programs for hematological malignancies remain concentrated on a small set of well-characterized surface antigens. CD19, CD22, BCMA, and CD33 are the antigens most frequently exploited in CAR-NK constructs directed against hematologic tumors, and bispecific formats pairing two of these markers such as CD19/BCMA, CD19/CD22, CD19/CD20, or BCMA/GPRC5D have been developed specifically to improve cytotoxicity against tumor populations with low or heterogeneous antigen density and to limit antigen-escape relapse (Balkhi et al., 2025; Liu et al., 2026). Parallel work in myeloid disease has centered on CD33, reflecting its prevalence on leukemic blasts, although this same marker is also expressed on normal hematopoietic progenitors, illustrating the narrow margin for target specificity in this indication (Li et al., 2025a; Albinger et al., 2022). This limited antigen landscape stems directly from discovery pipelines that identify “druggable” surface proteins almost entirely from transcriptomic or exomic data, an approach that overlooks a large portion of the actively translated proteome.

Ribosome profiling (Ribo-seq) offers a complementary discovery axis by sequencing the ribosome-protected RNA footprints generated during active translation, providing a direct, sequencing-based readout of the ribosome-bound fraction of the transcriptome as a proxy for protein synthesis, rather than an inference drawn from steady-state mRNA abundance (Ingolia et al., 2012). Because ribonuclease digestion of unprotected mRNA delineates the precise boundaries of each translated open reading frame (ORF), the method is particularly well suited to resolving small proteins that are systematically underrepresented in standard genome annotation because their features preclude reliable detection by conventional biochemical or computational approaches (Gelhausen et al., 2021). This feature is especially important for antigen discovery, because a large fraction of clinically useful peptides are not come from canonical, well-annotated genes. Multiple groups have shown that Ribo-seq can uncover classes of antigens that prediction pipelines based on transcriptome or exome data routinely overlook. In work on HLA class-I restricted minor histocompatibility antigens, ribosome profiling identified ORFs encoding cryptic antigen classes including those derived from upstream and alternative-transcript ORFs with substantially higher sensitivity than antigens arising from out-of-frame ORFs or long non-coding RNA–derived ORFs (Fuchs et al., 2025). In an independent tumor-focused application, Ribo-seq was used to identify thousands of peptides derived from novel, unannotated open reading frames (nuORFs) expressed across a panel of malignancies including melanoma, chronic lymphocytic leukemia, and glioblastoma, several of which were subsequently confirmed as *bona fide* MHC class I presented epitopes (Ouspenskaia et al., 2022).

For CAR-NK programs aimed at leukemias and lymphomas, this approach substantially widens the pool of candidate antigens beyond the canonical markers in current use. It also opens a path toward targets whose expression is more tightly confined to malignant cells, which could lower the risk of on-target, off-tumor toxicity against healthy hematopoietic cells. The practical value of Ribo-seq–derived candidates, however, hinges on orthogonal confirmation that a predicted ORF product is truly processed, trafficked, and displayed on the cell surface rather than degraded intracellularly. Mass spectrometry–based immunopeptidomics can validate the presence of the translated proteins and peptides nominated by Ribo-seq, providing direct biochemical evidence of protein expression and thereby strengthening confidence in the selected antigen candidates (Xavier-Magalhães et al., 2026). At scale, this integrative strategy has already yielded substantial results: a reanalysis combining publicly available immunopeptidomic data across fourteen tumor types and twenty-nine normal tissues with Ribo-seq–derived canonical and noncanonical ORF libraries identified 389,165 canonical and 70,270 noncanonical HLA-presented peptides, enabling direct comparison of tumor versus normal-tissue presentation to flag antigens with an acceptably narrow safety margin (Cai et al., 2025). This tumor-versus-normal comparison step is essential for CAR-NK target vetting, given that the field’s central design constraint remains identifying antigens that combine high specificity, broad coverage of the malignant clone, and stable expression criteria that few current targets satisfy simultaneously (Yang et al., 2024a).

Practically, Ribo-seq is compatible with the sample-scarcity constraints typical of hematological malignancy research. Gradient-free Ribo-seq protocols have extended profiling to as few as approximately 200 cells, making the method applicable to rare populations such as circulating tumor cells and FACS-sorted subsets (Tastsoglou et al., 2023), a feature that is particularly relevant for interrogating minimal residual disease clones or sorted leukemic stem and progenitor cell fractions that are otherwise difficult to obtain in bulk. On the analytical side, extracting confident ORF calls from raw footprint data requires dedicated computational tools; algorithms such as RibORF, RiboCode, RiboDIPA, ORFRater, RiboTaper, ORFscore, RP-BP, and PRICE are commonly used to score and prioritize small open reading frames identified from ribosome profiling data, and pipelines that couple these ORF-calling algorithms with proteogenomic validation have been developed specifically to maximize confidence in microprotein- and smORF-encoded candidates that would otherwise be missed by Ribo-seq analysis alone (Vieira De Souza et al., 2024).

Overall, these advances cast Ribo-seq as a complementary, not replacement layer in CAR-NK target-discovery workflows. It helps nominate candidate ORFs that transcriptomic screens miss, checks their translation against normal-tissue translatomes to gauge safety margins, and funnels the surviving candidates through immunopeptidomic and functional validation before moving on to single-chain variable fragment or NK-activating receptor design. As Ribo-seq protocols continue to lower input requirements and boost annotation confidence, integrating this method into the antigen-discovery arm of CAR-NK engineering programs is likely to become increasingly routine, especially for indications like acute myeloid leukemia where the canonical antigen repertoire remains particularly limited.

Although rapid improvements in genome engineering, synthetic biology, and AI-driven platforms for CAR-NK development are rapidly occurring, it is critical to distinguish between tantalizing preclinical observations and proven clinical demonstration. Most CRISPR-based CAR-NK engineering strategies and synthetic regulatory circuits have been evaluated primarily through *in vitro* studies and preclinical models, whereas clinical validation remains limited (Clubb et al., 2023). Similarly, AI-guided CAR-NK design is currently at early computational and experimental stages and requires further biological validation and clinical investigation. Therefore, although these technologies represent important opportunities for next-generation CAR-NK therapies, their safety, efficacy, and scalability require continued assessment before broad clinical implementation.

Despite these advances, AI-guided CAR-NK engineering remains limited by several important challenges. The performance of computational models depends strongly on the availability, size, and quality of training datasets, which are still relatively limited and heterogeneous in cellular immunotherapy. Moreover, the interpretability of complex AI models remains a major concern, as predictions generated by deep-learning approaches may not always provide clear biological explanations. Therefore, AI-driven predictions should be considered as supportive tools rather than replacements for experimental validation, requiring confirmation through functional assays and clinical studies before translation into therapeutic applications (Kim, 2025).

## 8 Clinical experience with CAR-NK cells in hematologic malignancies: response rates, durability, and safety across CD19^−^ and CD33-directed trials

Engineering advances in CAR-NK cell design have outpaced, in the literature, a disciplined accounting of what these constructs have actually achieved in patients. Here we summarize in Table 7 the clinical data generated to date across the major CD19^−^ and CD33-directed CAR-NK programs in hematologic malignancy, with particular attention to response durability and treatment-related toxicity.

**TABLE 7:** Summary of completed CAR-NK trials in hematologic malignancies.

Feature	Cord blood CD19 CAR-NK (phase 1/2, n = 11)	Cord blood CD19 CAR-NK (final, n = 37)	iPSC-derived CD19 CAR-NK, FT596 (n = 86)	Cord blood CD33 CAR-NK, AML (n = 10)
Target	CD19	CD19	CD19	CD33
Cell source	Cord blood–derived NK	Cord blood–derived NK	iPSC-derived NK	Cord blood–derived NK
Armoring	IL-15 + inducible caspase-9 safety switch	IL-15 + inducible caspase-9 safety switch	Not specified in source	Not specified in source
Lymphodepletion	Lymphodepleting chemotherapy	Lymphodepleting chemotherapy	Cyclophosphamide/fludarabine	Fludarabine-based preconditioning
Dose	1 × 10^5^, 1 × 10^6^, or 1 × 10^7^ cells/kg (escalating)	Same product/dosing framework	RP2D ∼1.8 × 109 cells (3 doses/cycle)	Not specified in source
N	11 (R/R NHL or CLL)	37 (CD19^+^ B-cell malignancies)	86 (R/R B-cell lymphoma; monotherapy n = 18, +rituximab n = 68)	10 (R/R AML)
Response rate	73% ORR (7 CR + 1 partial Richter’s clearance)	48.6% ORR at day 30 and day 100 (varied by histology: 100% low-grade NHL, 67% CLL, 41% DLBCL)	ORR not fully reported for full cohort; interim readout at highest dose: 8/11 responded (7 CR)	60% MRD-negative CR by day 28
Durability	Not yet established per accompanying commentary; cells persisted ≥12 months	1-yr OS 68%, 1-yr PFS 32% — durable control in ∼1 in 3	Described as “deep and durable” by investigators; mature (>1-yr) data not yet reported	Not yet established; investigators note longer follow-up needed
CRS/ICANS	None observed	1 patient, grade 1 CRS; no neurotoxicity	6% (mono)/13% (combo) CRS, grade ≤2; no neurotoxicity	1 patient, grade 2 CRS; no ICANS
GvHD	None observed	None observed	Not reported	None observed
Persistence	Low-level expansion/persistence ≥12 months	Higher persistence associated with response	Not fully reported	Not specified in source

FT596 armoring strategy, exact dosing regimen, and full-cohort response/durability figures, as well as CD33 CAR-NK, dosing and persistence data, were not specified in the sources available at the time of this revision.

### 8.1 Cord blood–derived CD19 CAR-NK cells in relapsed/refractory CD19^+^ lymphoid tumors

The earliest clinical data on this approach came from a phase 1/2 trial in which 11 patients with relapsed or refractory CD19-positive non-Hodgkin lymphoma or chronic lymphocytic leukemia were treated with cord blood–derived, anti-CD19 CAR-NK cells engineered to co-express IL-15 and an inducible caspase-9 safety switch. Each patient received lymphodepleting chemotherapy followed by a single infusion of these HLA-mismatched cells, given at one of three escalating dose levels 1 × 10^5^, 1 × 10^6^, or 1 × 10^7^ CAR-NK cells per kilogram (Marin et al., 2024). Eight of the 11 treated patients (73%) responded, with 7 achieving complete remission 4 with lymphoma and 3 with CLL and one further patient clearing the Richter’s transformation component of their disease while their underlying CLL remained. These responses came on quickly, showing up within 30 days regardless of dose level, and the infused cells went on to expand and persist at low levels for at least a year afterward. Just as strikingly, none of the patients developed cytokine release syndrome, neurotoxicity, or graft-versus-host disease, at any of the doses tested. An accompanying commentary on this trial cautioned that, while the initial efficacy and toxicity profile were favorable, the durability of these early responses could not yet be established from the reported follow-up (Karadimitris, 2020). We regard this caveat as essential to reproduce rather than omit, given how frequently early CAR-NK reports are cited for response rate alone.

### 8.2 The subsequent, larger phase 1/2 experience (final report, n = 37)

Building on this earlier work, the finalized trial went on to treat 37 patients with CD19-positive B-cell malignancies using the same cord blood–derived CAR19/IL-15 NK cell product. The safety picture held up: just as in the initial report, no meaningful toxicities emerged no cytokine release syndrome, no neurotoxicity, no graft-versus-host disease with a single case of grade 1 CRS the only exception across the whole cohort (Marin et al., 2024). Overall response rates held steady at 48.6% at both day 30 and day 100, though the picture changed considerably depending on histology every patient with low-grade non-Hodgkin lymphoma responded, compared with 67% of those with untransformed CLL and 41% of those with diffuse large B-cell lymphoma. At one-year, overall survival stood at 68%, while progression-free survival dropped to 32% (Marin et al., 2024). Together, these numbers tell a more sobering story than the response rate alone suggests: most patients are still alive at 1 year, but only about one in three actually have their disease durably controlled a gap that gets lost if you look at response rates in isolation.

Exploratory analyses offered a clue as to why some patients did better than others: those who responded had higher levels and longer-lasting persistence of CAR-NK cells in circulation. Interestingly, certain characteristics of the donor cord blood unit itself, namely, cryopreservation within 24 h of collection and a low nucleated red blood cell content predicted who would respond. That points to something worth emphasizing: in this allogeneic setting, it's not just the CAR construct that drives outcomes, but the quality of the raw starting material (Marin et al., 2024).

### 8.3 iPSC-derived CD19 CAR-NK cells (FT596) in B-cell lymphoma

FT596, an induced pluripotent stem cell–derived CD19 CAR-NK product, has been evaluated in a first-in-human trial enrolling 86 patients with relapsed or refractory B-cell lymphoma who had received a median of four prior lines of therapy, 38% with prior CAR-T cell exposure. Patients were treated with FT596 as monotherapy (n = 18) or in combination with rituximab (n = 68) following cyclophosphamide/fludarabine conditioning. The maximum tolerated dose was not reached, and the recommended phase 2 dose was preliminarily identified as 1.8 × 10^9^ cells given as three doses per cycle (Ghobadi et al., 2025). Cytokine release syndrome occurred in 6% of monotherapy patients (maximum grade 1) and 13% of combination-therapy patients (grade 1–2), with no neurotoxicity observed at any dose. The trial has been reported by its investigators as inducing “deep and durable responses” in both indolent and aggressive lymphoma, and an earlier interim readout at a single higher dose level (≥90 million cells) demonstrated an objective response in 8 of 11 efficacy-evaluable patients, including 7 complete responses. Notably, transparency that the aggregate overall response and complete response rates for the full 86-patient cohort by dose level, and mature (>1-year) durability data, were not fully reported in the sources available to us at the time of this revision. We have therefore worded this section to reflect “deep and durable” as the trial’s own characterization pending publication of long-term follow-up, rather than asserting a specific durability figure that our sources do not support.

### 8.4 Early CD33-directed CAR-NK data in acute myeloid leukemia

In a first-in-human phase 1 trial, cord blood–derived CD33 CAR-NK cells designed specifically to spare hematopoietic stem cells relative to CD33 CAR-T constructs were administered to 10 patients with relapsed/refractory AML (median 5 prior lines of therapy) as one or more infusions following fludarabine-based preconditioning. Six of 10 patients (60%) achieved MRD-negative complete remission by day 28 (Huang et al., 2025b). No grade 3–4 adverse events were observed other than transient bone marrow suppression that resolved within 1 month; no cases of immune-effector-cell-associated neurotoxicity syndrome or graft-versus-host disease were reported, and only one patient developed grade 2 cytokine release syndrome. The investigators themselves emphasize that expanded patient samples and longer follow-up are needed before efficacy and durability can be considered established (Huang et al., 2025b).

### 8.5 Leading CAR-NK programs: from hematologic malignancies to emerging solid tumor and autoimmune applications

CAR-NK cell therapy has advanced rapidly from early proof-of-concept studies to a diverse pipeline spanning multiple antigen targets and disease indications. Table 8 summarizes the current clinical and advanced preclinical landscape, highlighting lead programs from academic centers and biotech sponsors, their cell sources and engineering strategies, and their outcomes to date. While CD19-directed CAR-NK constructs, particularly cord blood-derived and iPSC-derived platforms, have generated the most mature clinical data, including encouraging response rates in relapsed/refractory B-cell malignancies, several programs have also been discontinued due to strategic or durability challenges rather than safety concerns. Emerging efforts targeting solid tumors and autoimmune diseases remain earlier in development but reflect the field’s expanding scope beyond hematologic malignancies.

**TABLE 8:** Current clinical and advanced preclinical landscape.

Product/Sponsor	Source/Design	Indication/Stage	Key outcome	Status
CD19 CAR-NK (MD Anderson/Rezvani lab)	Cord blood–derived; retroviral CD19-CAR + IL-15 + inducible caspase-9 safety switch	R/R CD19^+^ NHL/CLL; Phase 1/2 (n = 37, final report)	48.6% overall response at day 100; 1-yr PFS 32%, OS 68%; no severe CRS, neurotoxicity, or GvHD; early cohort showed 73% ORR/64% CR	Ongoing; expanding to new antigens/solid tumors
FT596 (Fate Therapeutics)	iPSC-derived, clonal master line; CD19 CAR + hnCD16 (ADCC) + IL-15RF	R/R B-cell lymphoma; Phase 1 (first-in-human iPSC CAR-NK)	High-dose repeat infusions (108–109 cells/dose) without reaching MTD; persistence to ∼15 days; meaningful response rates in combination with rituximab	Discontinued (2023, business/pipeline prioritization, not efficacy failure)
NKX101 (Nkarta)	Allogeneic, donor-derived; NKG2D-ligand-targeted CAR + membrane-bound IL-15	R/R AML/MDS; Phase 1	Early cohort: 67% CR/CRi with Flu/Ara-C conditioning; expanded cohort: response rate fell to ∼25%	Discontinued (2024) after loss of durable response on expansion
NKX019 (Nkarta)	Peripheral-blood-derived; CD19 CAR + membrane-bound IL-15	R/R B-cell malignancies; Phase 1, now also autoimmune disease (Ntrust-1/2)	70% CR rate at higher dose levels in early cohorts; lymphodepletion regimen since optimized (Flu + Cy) for complete B-cell depletion	Active; pivoting toward autoimmune indications
QN-139b (academic/biotech collaboration)	iPSC-derived; dual CD19/BCMA CAR, gene-edited for reduced alloreactivity	Severe systemic sclerosis (autoimmune); first-in-human case	Marked clinical response with significant B-cell depletion and minimal toxicity; no chromosomal abnormalities detected	Early-stage; single-patient proof-of-concept
CD33 CAR-NK	NK-92-based and primary-cell-based constructs	R/R AML; Phase 1 and preclinical	Preclinical efficacy confirmed; early-phase trial data support safety and target engagement	Early clinical stage
CAR-NK for solid tumors (ROBO1, CEA, mesothelin, HER2, GD2, etc.)	Primary or NK-92-based; various costimulatory/cytokine armoring	Pancreatic, colorectal, ovarian, glioblastoma, other solid tumors; largely preclinical/early Phase 1	Encouraging preclinical cytotoxicity; clinical responses so far modest and inconsistent, limited by trafficking, persistence, and TME suppression	Preclinical to early clinical

### 8.6 Addressing durability

In the original 11-patient cord blood CD19 CAR-NK trial, an accompanying editorial explicitly cautioned that favorable initial efficacy and toxicity findings did not yet establish durability of clinical responses, and several responding patients subsequently underwent consolidative stem cell transplantation, making it difficult to attribute long-term remission to the CAR-NK product in isolation (Rezvani, 2025). In the larger, completed 37-patient trial, the gap between one-year overall survival (68%) and one-year progression-free survival (32%) is itself the clearest quantitative statement of limited durability available for this platform: most patients are alive at 1 year, but only about one-third remain progression-free, indicating that a majority of initial responses are not sustained at the one-year landmark. For FT596, the trial’s own descriptive language (“deep and durable”) is not yet substantiated in the literature available to us by a published aggregate durability metric (e.g., median duration of response or 1-year PFS) for the full 86-patient cohort, and we have avoided implying otherwise. For the CD33 AML data, the cohort is small (n = 10), and follow-up is explicitly described by the investigators as too short to draw durability conclusions.

## 9 Future perspectives

The potential evolution of CAR-NK therapy will not only include enhanced cytotoxic effects but also provide cells with the ability to recognize their environment, change their functionality, and self-regulate. For instance, the response of CAR-NK products in hematological malignancies should be based on features such as antigen density, degree of marrow suppression, presence of cytokines, and signals of relapse rather than purely on killing fixed targets. Ultimately, this new direction will result in CAR-NK cells being viewed as ‘programmable therapeutic agents’ instead of simply engineered immune cells (Zhao et al., 2023; Biarnes Carrera et al., 2024).

The *in vivo* CAR-NK engineering approach is a new method that has the potential to streamline both the production and application processes of CAR-NK therapy. Instead of obtaining NK cells, modifying and expanding them *in vitro*, and finally giving back the modified product in infusion form, the method utilizes targeted delivery systems that allow the use of CAR-encoding mRNA, gene editing components, or other genetic carriers to deliver these components or payloads directly to NK cells (Li et al., 2025d). Utilizing this method could lead to fewer issues with efficiency and logistics in production and delivery of CAR-NK cells. The use of targeted nanoparticles and other types of cell-targeting delivery technologies may help in this situation. Still, ensuring effective and selective delivery proves to be a complex task. The challenges of this method include the issue of controlling the effect of CAR expression in NK cells, its efficiency and its duration, and off-target modifications (Golubovskaya et al., 2023).

Transient, non-viral approaches for modifying NK-cell function without permanent genomic inserts will also provide valuable options for NK-cell engineering. Lipid nanoparticles can be used to deliver cytokine mRNA to NK cells; this enhances the ability of NK cells to proliferate and kill target cells, connecting traditional methods of manufacturing cells with novel, programmable approaches to *in vivo* modulation. This option is particularly appealing when using temporary assistance is safer than using permanent receptors or cytokines in NK cells (Delehedde et al., 2024; Mahalingam et al., 2024).

A complete vision is a completely independent immune system capable of detecting diseases and then initiating an appropriate response to those diseases based on an analysis of the overall context until the malignant signal is no longer present. Current technologies that regulate external controls and provide stimulus-responsive synthetic systems demonstrate how engineered cells can potentially be controlled/performed in real-time as therapeutics, thereby providing a complete vision through CAR-NK therapy and autonomous control bounded by predictable safety and risk, reversible means of control over those therapies, and clear clinical outcomes (Lin Z. et al., 2025; Monck et al., 2024).

## 10 Conclusion

CAR-NK cell therapy has rapidly progressed from a conventional antigen-targeting strategy toward a more sophisticated and adaptable form of cellular immunotherapy. Although early-generation platforms have demonstrated favorable safety profiles and promising antitumor activity, their clinical effectiveness remains limited by biological barriers such as antigen escape, functional exhaustion, and the immunosuppressive tumor microenvironment. These challenges highlight the need for fundamentally new design principles rather than incremental improvements to existing CAR architecture.

The next phase of CAR-NK development will be shaped by the convergence of multiple advanced disciplines. Genome editing technologies provide precise control over cytotoxic potency, persistence, and resistance to immune inhibition. Synthetic biology introduces programmable cellular circuits that enable logic-gated recognition, context-dependent activation, and controlled effector responses. Epigenetic engineering adds an additional regulatory layer, allowing reversible modulation of transcriptional programs associated with exhaustion, persistence, and functional fitness. In parallel, multiomic technologies are redefining target discovery by capturing tumor heterogeneity at single-cell and spatial resolution, while artificial intelligence integrates these complex datasets into predictive models that guide receptor design and therapeutic optimization.

The future of CAR-NK therapy will not be determined solely by the discovery of new tumor antigens, but by the integration of genome editing, synthetic biology, epigenetic regulation, multiomics, and artificial intelligence into a unified framework for precision cellular engineering. This convergence will enable the development of adaptive, patient-specific, and functionally resilient CAR-NK systems capable of overcoming tumor evolution and achieving durable clinical responses in hematologic malignancies.
